# Physiological effects and safety of bed verticalization in patients with acute respiratory distress syndrome

**DOI:** 10.1186/s13054-024-05013-y

**Published:** 2024-08-05

**Authors:** Louis Bouchant, Thomas Godet, Gauthier Arpajou, Lucie Aupetitgendre, Sophie Cayot, Renaud Guerin, Matthieu Jabaudon, Camille Verlhac, Raiko Blondonnet, Lucile Borao, Bruno Pereira, Jean-Michel Constantin, Jean-Etienne Bazin, Emmanuel Futier, Jules Audard

**Affiliations:** 1 Department of Perioperative Medicine, Centre Hospitalier Universitaire (CHU) Clermont-Ferrand, 1 Place Lucie Et Raymond Aubrac, 63000 Clermont-Ferrand, France; 2 Department of Healthcare Simulation, Université Clermont Auvergne, Clermont-Ferrand, France; 3 Université Clermont Auvergne, iGreD, CNRS, INSERM, Clermont-Ferrand, France; 4 Direction de la Recherche Clinique et de l’Innovation (DRCI), Centre Hospitalier Universitaire (CHU) Clermont-Ferrand, Biostatistics Unit, Clermont-Ferrand, France; 5 Assistance Publique-Hôpitaux de Paris (AP-HP), Département Anesthésie et Réanimation, Hôpital Pitié-Salpêtrière, DREAM, Sorbonne Université, Paris, France

**Keywords:** Acute respiratory distress syndrome, Verticalization, Intensive care unit, Mechanical ventilation

## Abstract

**Background:**

Trunk inclination in patients with Acute Respiratory Distress Syndrome (ARDS) in the supine position has gained scientific interest due to its effects on respiratory physiology, including mechanics, oxygenation, ventilation distribution, and efficiency. Changing from flat supine to semi-recumbent increases driving pressure due to decreased respiratory system compliance. Positional adjustments also deteriorate ventilatory efficiency for CO_2_ removal, particularly in COVID-19-associated ARDS (C-ARDS), indicating likely lung parenchyma overdistension. Tilting the trunk reduces chest wall compliance and, to a lesser extent, lung compliance and transpulmonary driving pressure, with significant hemodynamic and gas exchange implications.

**Methods:**

A prospective, pilot physiological study was conducted on early ARDS patients in two ICUs at CHU Clermont-Ferrand, France. The protocol involved 30-min step gradual verticalization from a 30° semi-seated position (baseline) to different levels of inclination (0°, 30°, 60°, and 90°), before returning to the baseline position. Measurements included tidal volume, positive end-expiratory pressure (PEEP), esophageal pressures, and pulmonary artery catheter data. The primary endpoint was the variation in transpulmonary driving pressure through the verticalization procedure.

**Results:**

From May 2020 through January 2021, 30 patients were included. Transpulmonary driving pressure increased slightly from baseline (median and interquartile range [IQR], 9 [5–11] cmH_2_O) to the 90° position (10 [7–14] cmH_2_O; *P* < 10^–2^ for the overall effect of position in mixed model). End-expiratory lung volume increased with verticalization, in parallel to decreases in alveolar strain and increased arterial oxygenation. Verticalization was associated with decreased cardiac output and stroke volume, and increased norepinephrine doses and serum lactate levels, prompting interruption of the procedure in two patients. There were no other adverse events such as falls or equipment accidental removals.

**Conclusions:**

Verticalization to 90° is feasible in ARDS patients, improving EELV and oxygenation up to 30°, likely due to alveolar recruitment and blood flow redistribution. However, there is a risk of overdistension and hemodynamic instability beyond 30°, necessitating individualized bed angles based on clinical situations.

**Trial registration** ClinicalTrials.gov registration number NCT04371016, April 24, 2020.

**Supplementary Information:**

The online version contains supplementary material available at 10.1186/s13054-024-05013-y.

## Background

Mobilization of patients with Acute Respiratory Distress Syndrome (ARDS) is a subject of considerable inquiry. While prone positioning has provided some answers, trunk inclination remains a central question that continues to attract increasing scientific interest due to its notable effects on respiratory physiology. Indeed, changes in inclination angles significantly influence respiratory mechanics, oxygenation, ventilation distribution, and ventilatory efficiency [1–5].

Although changing the position from flat supine to semi-recumbent has shown an improvement in oxygenation in certain patients, particularly those with an increase in end-expiratory lung volume (EELV) [3, 4], research has primarily revealed an increase in driving pressure (ΔP), reflecting a decrease in respiratory system compliance (C_RS_) [1, 2]. Additionally, a reduction in carbon dioxide (CO_2_) elimination has been observed, suggesting probable overdistension.

A more precise analysis of compliance variations reveals that the decrease is mainly in chest wall compliance (C_CW_) and, to a lesser extent, lung compliance (C_L_). These physiological data are particularly significant because C_CW_ alterations are associated with adverse hemodynamic effects, whereas C_L_ alterations primarily affect gas exchange [6, 7]. These effects can be explained by an increase in intra-abdominal pressure during the transition from the flat supine to the semi-recumbent position [8–10]. The 45° reverse-Trendelenburg position without hip flexion can exacerbate this increase in intra-abdominal pressure due to a more significant shift of abdominal contents toward the bladder, influenced by gravity, compared to the semi-recumbent and flat supine positions [11, 12]. However, the impact of this increase in intra-abdominal pressure caused by the downward displacement of abdominal organs on intrathoracic pressures remains uncertain. Therefore, the effect of bed verticalization on cardio-pulmonary physiological changes needs to be determined.

The primary objective of this study was to investigate whether progressive verticalization without hip flexion in patients undergoing mechanical ventilation can improve oxygenation while minimizing its impact on respiratory mechanics, as represented by the variation in transpulmonary driving pressure (ΔP_L_). The secondary objectives were to evaluate changes in pulmonary and cardiovascular physiology and to assess the intervention's feasibility and safety.

## Methods

For detailed information on patient installation, technics used (pulmonary artery catheter zero reference setting, EELV measurement by nitrogen washout/washin, dead space and shunt measurements) and their limitations, please refer to Appendix Files in electronic supplementary file (Supplementary Material 1: Appendix File 2).

### Study design

This study was a prospective, pilot physiological study in which each patient was their own control. It was conducted in two intensive care units at CHU Clermont-Ferrand (France). The trial was approved by the French ethics committee (*Comité de Protection des Personnes Ouest IV Nantes*) on July 2, 2019, and the medicine agency (EudraCT 2019-A01006-51). The protocol was registered on ClinicalTrials.gov (NCT04371016).

### Study population

Adult patients admitted to participating ICUs with early (< 12 h) moderate-to-severe ARDS according to the Berlin criteria [13] and receiving invasive, controlled mechanical ventilation through an endotracheal tube or a tracheostomy, deep sedation, and neuromuscular blockade were eligible. Obese (body mass index [BMI] ≥ 35 kg m^−2^) and hemodynamically unstable patients (as defined by an increase of > 20% in catecholamine requirements in the last hour, despite blood volume optimization to reach a mean arterial pressure target of 65–75 mmHg), were not included.

### Patient installation

**Fig. 1:**
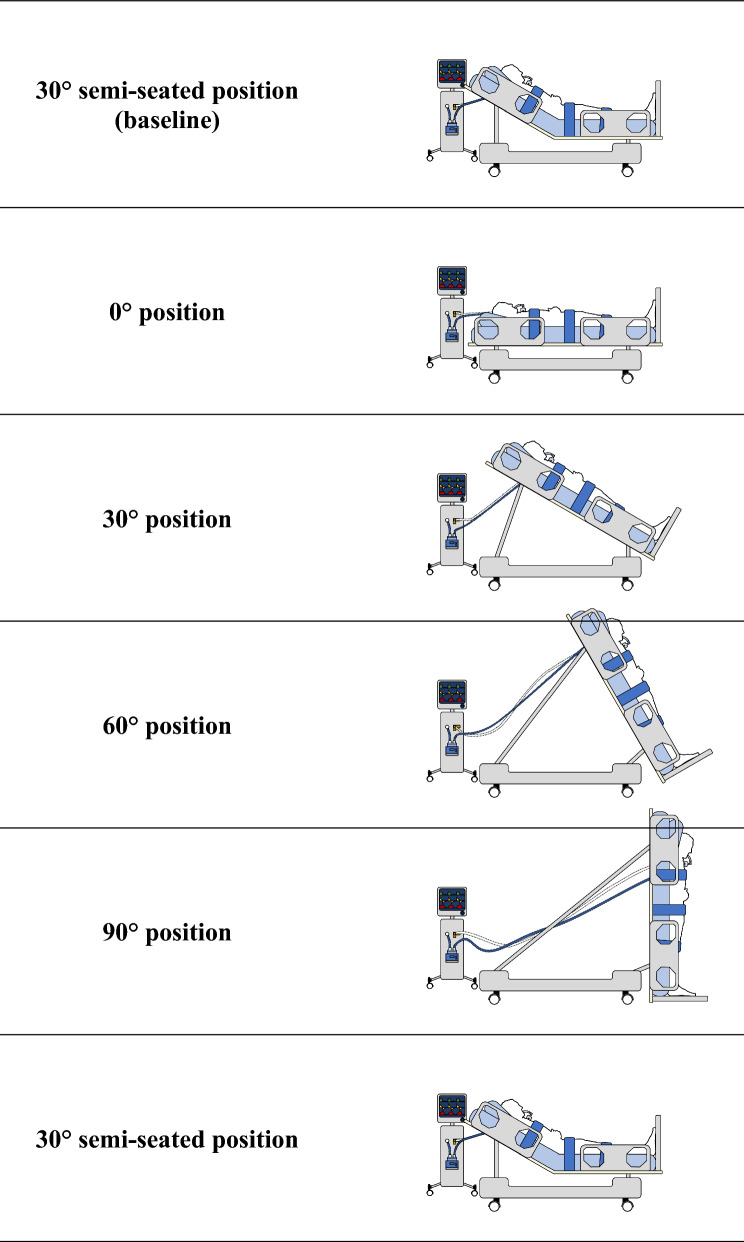
Diagram of the progressive verticalization procedure. All patients started the sequence in the 30° semi-seated position (baseline). After 30 min, successive steps at 0°, 30°, 60°, and 90° were reached for 30 min each, allowing progressive bed verticalization without body flexion (standing upright). Then, patients were returned to the 30° semi-seated position for 30 min

A verticalization team was called each time a patient was deemed eligible by the treating physician. Recent technological advances in the field of critical care beds, such as the Total Lift Bed™ (VitalGo Systems Inc., Arjo AB, Illinois, USA), now allow the easy positioning of patients in a standing, upright position (up to 90°) without any body flexion. After eligibility and consent were checked, patients were placed on a dedicated bed and secured with three large flexible straps at the knee, pelvis, and medio-thoracic levels (please see Supplementary Material 1: Appendix File 1). A pulmonary artery catheter (PAC) was inserted, for continuous hemodynamic monitoring (please see Supplementary Material 1: Appendix File 3). Tidal volume was set at 6 mL kg^−1^ of predicted body weight (PBW) in volume control mode and remained unchanged throughout the experiment. Positive end-expiratory pressure (PEEP) was titrated after alveolar recruitment maneuver (e-sigh in pressure control mode with inspiratory pressure of 10 cmH_2_O, respiratory rate of 20 bpm and stepwise increase of PEEP every 10 cycles with steps of 5 cmH_2_O (reaching maximum pressure of 50 cmH_2_O), with decremental steps of PEEP in volume control mode (6 mL kg^−1^ of PBW), in order to minimize driving pressure (ΔP = P_plat_-PEEP). An esophageal balloon catheter was inserted, and its correct position was confirmed using the Baydur method [14]. To limit measurement errors related to the displacement of the mediastinum structures and the redistribution of tidal volume within the thorax, we used the Baydur method to confirm the correct position of the esophageal catheter at each change of position, which is essential for obtaining accurate measurements of pleural pressure (Supplementary Material 1: Appendix File 1). After securing the patient, the bed could be gradually verticalized from 0° to 90° without body flexion, ensuring a standing upright position (Fig. 1).

### Positioning, equipment and measurements

The reference position was defined when patients were in a semi-seated position at 30°, considered as the baseline position. We established the zero reference of the respiratory and hemodynamic measurement systems during patient verticalization with this position, which appears to be the most common among patients undergoing mechanical ventilation. Furthermore, it appears to reduce the incidence of ventilator-associated pneumonia compared to the strictly supine position at 0° [15]. All patients started the sequence in a 30° semi-seated position (baseline). After 30 min, 30-min-long successive steps at 0°, 30°, 60°, and 90° were performed, allowing progressive bed verticalization without body flexion. Then, patients were returned to the 30° semi-seated position for 30 min (Fig. 1).

To limit the risk of low blood pressure during verticalization, all patients were continuously monitored using PAC. Fluid optimization was managed based on preload dependency data (Supplementary Material 1: Appendix File 1) [16]. In case of hemodynamic instability, the patient was positioned back to the previous step. At the end of each 30-min step, identical complete sets of data were collected, including hemodynamic data with PAC, ventilatory parameters, EELV, esophageal pressures, and blood gases parameters.

Parameters corresponding to primary and secondary outcomes were calculated as described below. ΔP_L_ was computed as ΔP-ΔP_es_, where ΔP_es_ was the difference between the end-inspiratory (measured during an end-inspiratory pause of 3 s) and the end-expiratory (measured during an end-expiratory pause of 5 s) esophageal pressure (P_es_) and ΔP was the driving pressure, computed as the difference between the inspiratory plateau pressure (P_plat_, measured during an end-inspiratory pause of 3 s) and the total level of PEEP (measured during an end-expiratory pause of 5 s). EELV was measured using the nitrogen washin/washout method (Supplementary Material 1: Appendix File 2) on Engström Carestation™ or Carescape ™ R860 ventilators, General Electric Healthcare, USA) [17]. Pulmonary dead-space fraction (V_D_/V_T_) as calculated using a rearranged alveolar equation for PaCO_2_ with modified Harris-Benedict equation to estimate the CO_2_ production [18].

### Outcomes

The primary endpoint was the variation in ΔP_L_ (as measured at the end of each verticalization step) between the baseline, 0°, 30°, 60°, 90° positions, and return to the 30° semi-seated position.

Secondary endpoints included the variations in the following parameters between the baseline, 0°, 30°, 60°, 90° positions, and return to the 30° semi-seated position: maximal transpulmonary pressure (alveolar stress), alveolar strain (V_T_/EELV), ΔP, pulmonary dead-space fraction (V_D_/V_T_), static compliance of the respiratory system (C_RS_, computed as V_T_/ΔP, lung compliance (C_L_, calculated as V_T_/ΔP_L_), chest wall compliance (C_CW_, computed as V_T_/ΔP_es_, pulmonary shunt fraction (using the Berggren equation) [19], mechanical power, hemodynamic parameters (heart rate, systemic and pulmonary arterial blood pressures, cardiac output, stroke volume, systemic and pulmonary vascular resistances, pulmonary artery occlusion pressure, arterial and mixed venous blood gas parameters (PaO_2_, PaCO_2_, SaO_2_, SvO_2_), and adverse events possibly related to verticalization(Additional File 1**)**.

### Safety endpoints

Safety endpoints and management are detailed in Supplementary Material 1: Appendix File 4. In case of inadvertent serious side effect occurrence, patient will be placed back in baseline position (30° semi-recumbent position) and side effect managed according to clinical habits and caracteristics.

### Statistical analysis

To demonstrate an effect size of 0.8 in transpulmonary driving pressure between the baseline, 0°, 30°, 60°, 90° positions and return to the 30° semi-seated position, the number of required subjects was estimated to be 30 early ARDS patients for a two-sided type I error rate of 2.5% (correction due to multiple comparisons), a statistical power greater than 80%, and an intra-individual correlation coefficient of 0.5 (correlation due to multiple positions evaluated in the same patient).

All results were compared according to the positions using mixed effects models, with time and patient position, respectively as fixed and random effect. The normality of residuals was analyzed using the Shapiro–Wilk test. When appropriate, a logarithmic transformation of dependent variable was applied to achieve the normality. For two by two positions comparisons, a Tukey–Kramer test was performed when omnibus p-value was less than 0.05. The relationships between changes at 0, 30, 60 and 90 were analyzed using Spearman correlation coefficients applying a Sidak’s type I correction. A two-sided p-value of less than 0.05 was considered to indicate statistical significance. More detailed statistical analyses are available in the supplementary materials (Supplementary Material 1: Appendix File 1).

## Results

### Baseline characteristics of subjects

From May 2020 through January 2021, a total of 30 patients were included. Their main characteristics upon study enrollment are presented in Table 1.

**Table 1:** Characteristics of patients

Characteristic	Study cohort (N = 30)
Demographic characteristics	
Age, years	67 ± 10
Male sex, n (%)	23 (77)
BMI, kg.m^−2^	29 ± 5
Medical respiratory history*, n (%)	8 (27)
SAPS II	43 ± 12
Length of mechanical ventilation prior to enrolment, days	2.6 ± 2.6
COVID-19 ARDS, n (%)	22 (73)
Ventilatory parameters	
PEEP, cmH_2_O	12 ± 2
Tidal volume, mL kg^−1^ PBW	6.5 ± 0.6
Respiratory rate, min^−1^	26 ± 4
P_plat_, cmH_2_O	24 ± 3
C_RS_, mL.cmH_2_O^−1^	40 ± 13
Driving pressure, cmH_2_O	12 ± 4
PaO_2_, mmHg	73 ± 19
PaO_2_/FiO_2_, mmHg	113 ± 34
PaCO_2_ mmHg	48 ± 11
FiO_2_, %	68 ± 16

Values are reported as mean ± standard deviation (SD) or n (%). Body mass index (BMI) is the weight in kilograms divided by the square of the height in meters. SAPS II: simplified acute physiology score II. COVID-19: Coronavirus disease 2019. ARDS: acute respiratory distress syndrome. PEEP: positive end-expiratory pressure. PBW: predicted body weight. P_plat_: end-inspiratory plateau pressure. C_RS_: static compliance of respiratory system. PaO_2_: arterial partial pressure of oxygen. FiO_2_: fraction of inspired oxygen. PaCO_2_: arterial partial pressure of carbon dioxide

^*^Described as history of chronic obstructive pulmonary disease (COPD), asthma, pulmonary emphysema, interstitial lung disease, lung cancer, or severe pneumonia leading to hospitalization

### Primary endpoint

**Fig. 2:**
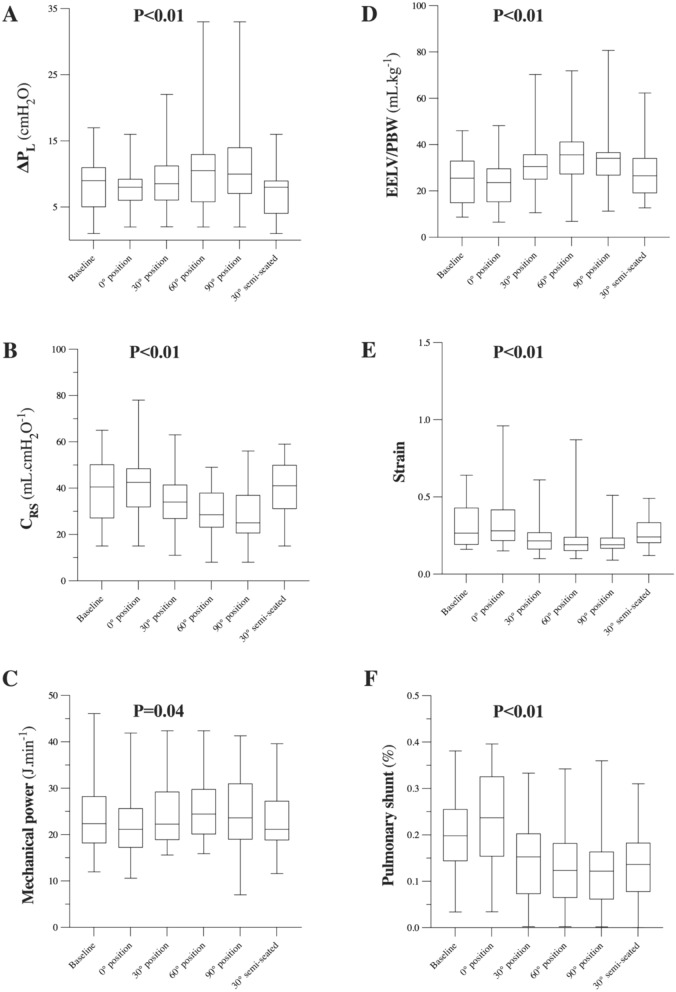
Physiological values evaluated at each position, from baseline (30° semi-seated position) to the standing upright position (90°), and a repositioning to 30° semi-seated. Values are reported as box and whisker plots. **A** Transpulmonary driving pressure (ΔP_L_), computed as the difference between end-inspiratory and end-expiratory transpulmonary pressures, as measured using an esophageal balloon catheter. **B** Static compliance of the respiratory system (C_RS_). **C** Mechanical power in the different study steps. **D** End-expiratory lung volume, as expressed in milliliters per kilogram of predicted body weight (EELV_PBW_). **E** Strain, computed as the tidal volume-to-EELV ratio. **F** Pulmonary shunt measured using the venous-to-arterial difference in oxygen concentrations. Mixed-effects models were used and the overall P value for the effect of position through the experimental procedure is provided

ΔP_L_ increased slightly during verticalization (P for the overall effect of position in mixed model < 10^–2^ (Fig. 2 and Table 2). The effect sizes are as follows: Baseline versus 0°: − 0.02 [− 0.38; 0.34], Baseline versus 30°: − 0.11 [− 0.46; 0.25], Baseline versus 60°: − 0.52 [− 0.88; − 0.16] (*P* = 0.004), Baseline versus 90°: − 0.61 [− 0.97; − 0.25] (*P* = 0.001), and Baseline vs end: 0.41 [0.06; 0.77] (*P* = 0.023). In post-hoc between-position comparisons, ΔP_L_ was higher in the straight 60° (median [interquartile range], 11 [6; 13] cmH_2_O) and 90° (10 [7; 14] cmH_2_O) positions than at baseline (semi-recumbent at 30°) (9 [5; 11] cmH_2_O) (*P* = 0.01 and *P* = 0.004, respectively), whereas it was lower in the final semi-recumbent at 30° position (8 [4; 9] cmH_2_O) compared to baseline (semi-recumbent at 30°) (*P* = 0.02).

**Table 2:** Physiological characteristics of ARDS according to verticalization steps

*Hemodynamic Status*	Baseline	0° position	30° position	60° position	90° position	30° semi-seated	*P* value
MAP, mmHg	76 (71; 80)	80 (74; 84)	75 (71; 79)	71 (68; 78)	74 (68; 81)	80 (74; 89)	< 0.01*
Heart rate, min^−1^	83 (71; 102)	83 (68; 96)	80 (72; 100)	88 (78; 113)	96 (88; 113)	91 (76; 106)	< 0.01*
Stroke volume, mL	72 (53; 89)	80 (60; 95)	72 (53; 93)	55 (42; 66)	48 (31; 65)	54 (42; 87)	< 0.01*
EDV, mL	151 (128; 220)	150 (132; 209)	166 (118; 222)	157 (112; 202)	150 (101; 183)	150 (120; 196)	0.13
CO, mL min^−1^	6.4 (4.5; 7.6)	6.5 (4.8; 8.0)	5.3 (4.3; 7.5)	4.9 (3.9; 6.2)	4.8 (3.2; 5.8)	4.5 (3.9; 6.7)	< 0.01*
RAP, mmHg	11 (9; 13)	12 (9; 14)	8 (6; 10)	5 (3; 7)	4 (3; 8)	10 (9; 13)	< 0.01*
SVR, mmHg min^−1^ mL^−1^	812 (654; 1201)	876 (652; 1194)	844 (709; 1249)	1152 (837; 1449)	1235 (898; 1730)	1058 (746; 1741)	< 0.01*
SvO_2_, %	67 (65; 76)	69 (62; 74)	69 (63; 76)	67 (56; 74)	65 (56; 76)	73 (66; 80)	< 0.01*
PAPm, mmHg	28 (25; 30)	29 (26; 30)	27 (25; 29)	27 (24; 28)	26 (22; 29)	29 (22; 31)	< 0.01*
PVR, mmHg min^−1^ mL^−1^	193 (143; 294)	181 (143; 266)	211 (161; 323)	271 (226; 402)	287 (241; 429)	250 (178; 309)	0.02*
PAOP, mmHg	11 (10; 13)	12 (11; 14)	10 (8; 12)	8 (7; 10)	7 (6; 9)	11 (10; 13)	< 0.01*
Serum lactate, mmol L^−1^	1.3 (1.1; 1.7)	1.3 (1.1; 1.7)	1.4 (1.2; 1.7)	1.5 (1.2; 1.9)	1.4 (1.2; 2.0)	1.4 (1.1; 1.8)	0.04*
Norepinephrine, µg kg^−1^ min^−1^	0.19 (0.07; 0.30)	0.19 (0.07; 0.29)	0.18 (0.10; 0.35)	0.27 (0.15; 0.46)	0.32 (0.16; 0.77)	0.21 (0.13; 0.45)	< 0.01*

*Respiratory variables*	Baseline	0° position	30° position	60° position	90° position	30° semi-seated	*P* value
PEEP, cmH_2_O	12 (10; 14)	12 (10; 14)	12 (10; 14)	12 (10; 14)	12 (10; 14)	12 (10; 14)	0.41
Tidal volume, mL kg^−1^ PBW	6.4 (6.1; 6.8)	6.5 (6.1; 6.8)	6.5 (6.1; 6.9)	6.5 (6.2; 6.8)	6.6 (6.1; 6.9)	6.5 (6.1; 7.0)	0.18
Respiratory rate, min^−1^	26 (24; 28)	26 (24; 28)	26 (22; 28)	26 (24; 28)	26 (24; 26)	26 (24; 26)	0.24
P_plat_, cmH_2_O	24 (23; 27)	24 (22; 25)	26 (23; 29)	28 (26; 31)	28 (26; 33)	24 (22; 25)	< 0.01*
C_RS_, mL cmH_2_O^−1^	41 (27; 50)	43 (32; 49)	34 (27; 42)	29 (23; 38)	25 (21; 37)	41 (31; 50)	< 0.01*
C_L_, mL cmH_2_O^−1^	47 (39; 84)	56 (43; 71)	53 (36; 80)	39 (32; 74)	43 (30; 63)	59 (44; 108)	0.11
C_CW_, mL cmH_2_O^−1^	117 (95; 178)	145 (113; 204)	90 (74; 126)	81 (65; 104)	81 (59; 96)	113 (86; 150)	< 0.01*
Driving pressure, cmH_2_O	11 (9; 14)	11 (9; 13)	13 (10;16)	14 (12; 19)	15 (12; 20)	11 (9; 13)	< 0.01*
ΔP_L_, cmH_2_O	9 (5; 11)	8 (6; 9)	9 (6; 11)	11 (6;13)	10 (7; 14)	8 (4; 9)	< 0.01*
Mechanical power, J min^−1^	22 (18; 28)	21 (17; 26)	22 (19; 29)	24 (20; 30)	24 (19; 31)	21 (19; 27)	0.04*
EELV_PBW_, mL kg^−1^	26 (15; 33)	24 (15; 30)	31 (25; 36)	36 (27; 41)	34 (27; 37)	27 (19; 34)	< 0.01*
V_D_/V_T_, %	69 (60; 77)	70 (62; 77)	73 (62; 79)	73 (66; 78)	73 (65; 81)	70 (60; 77)	0.16
Strain	0.27 (0.19; 0.43)	0.28 (0.22; 0.42)	0.22 (0.16; 0.27)	0.19 (0.15; 0.24)	0.19 (0.17; 0.24)	0.24 (0.20; 0.34)	< 0.01*
PaO_2_, mmHg	69 (62; 81)	61 (57; 73)	78 (69; 95)	81 (69; 101)	84 (68; 105)	73 (70; 87)	< 0.01*
PaO_2_/FiO_2_, mmHg	116 (84; 140)	96 (78; 124)	137 (99; 158)	142 (106; 192)	136 (113; 186)	113 (93; 156	< 0.01*
PaCO_2_, mmHg	47 (42; 52)	45 (37; 50)	48 (40; 53)	51 (42; 60)	51 (42; 60)	49 (41; 59)	< 0.01*
FiO_2_, %	65 (54; 80)	65 (54; 80)	65 (54; 80)	60 (50; 73)	60 (50; 75)	70 (50; 90)	0.33
FeCO_2_, %	1.59 (1.36; 1.90)	1.61 (1.25; 1.98)	1.52 (1.09; 2.00)	1.52 (1.34; 1.93)	1.49 (1.10; 1.82)	1.65 (1.23; 1.92)	0.08
VCO_2_, mL min^−1^	169 (134; 199)	174 (139; 193)	169 (133; 191)	169 (149; 193)	169 (124; 193)	172 (146; 196)	0.14
V_T_CO_2_, mL	6.9 (5.3; 8.2)	6.9 (4.8; 8.1)	6.8 (4.3; 7.7)	6.8 (5.6; 7.4)	6.9 (4.2; 7.4)	6.9 (4.7; 8.3)	0.17
Pulmonary shunt, %	20 (14; 26)	24 (15; 33)	15 (7; 20)	12 (6; 18)	12 (6; 16)	14 (8; 18)	< 0.01*
Arterial pH	7.33 (7.29; 7.40)	7.33 (7.28; 7.40)	7.33 (7.29; 7.38)	7.31 (7.25; 7.34)	7.30 (7.22; 7.32)	7.26 (7.31; 7.34)	< 0.01*
Serum bicarbonate, mmol L^−1^	26 (20; 30)	26 (21; 29)	26 (20; 31)	26 (19; 29)	25 (19; 28)	25 (22; 29)	< 0.15

Data are presented as median [25th–75th percentiles] unless otherwise indicated. Mixed-effects models were used for all variables, and the global P-values for position effect throughout the experimental procedure are provided. MAP: mean arterial pressure. EDV: end-diastolic volume. CO: cardiac output. RAP: right atrial pressure. SVR: systemic vascular resistance. SvO_2_: oxygen saturation of mixed venous blood. PAPm: mean pulmonary artery pressure. PVR: pulmonary vascular resistance. PAOP: pulmonary artery occlusion pressure. PEEP: positive end-expiratory pressure. P_plat_: end-inspiratory plateau pressure. C_RS_: static compliance of respiratory system. C_L_: lung compliance. C_CW_: chest wall compliance. ΔP_L_: transpulmonary driving pressure. EELV: end-expiratory lung volume. V_D_/V_T_: dead-space fraction. PBW: predicted body weight. PaO_2_: arterial partial pressure of oxygen. FiO_2_: fraction of inspired oxygen. PaCO_2_: arterial partial pressure of carbon dioxide

### Secondary endpoints

#### Respiratory mechanics

C_RS_ and C_CW_ deacreased significantly during the experimental procedure (P for the overall effect of position in mixed model < 10^–2^ for both variables), while C_L_ remained stable (P for the overall effect of position in mixed model = 0.11) (Fig. 2 and Table 2). EELV, as expressed in milliliters per kilogram of predicted body weight (EELV_PBW_), changed significantly during verticalization (P for the overall effect of position in mixed model < 10^–2^, with significant increase from baseline (semi-recumbent at 30°) to the straight 30°, 60°, and 90° positions (*P* < 10^–2^ for all post-hoc comparisons) (Table 2). There were significant changes in alveolar strain during the procedure (*P* for the overall effect of position in mixed model < 10^–2^, with decreases from baseline (semi-recumbent at 30°) to the straight 30°, 60°, and 90° positions (*P* < 10^–2^, *P* = 0.01, and *P* < 10^–2^, respectively), in mechanical power (*P* for the overall effect of position in mixed model = 0.04), although significant changes in the latter were found, in post-hoc analyses, only between the straight 0° and 60° positions (*P* < 10^–2^) and the 60° and final semi-recumbent at 30° positions (*P* = 0.02) (Fig. 2 and Table 2). There were no changes in V_D_/V_T_ during the verticalization procedure (*P* for the overall effect of position in mixed model = 0.16). Other respiratory variables are reported in Table 2.

#### Hemodynamic assessments

Mean arterial pressures significantly changed through the procedure (*P* for the overall effect of position in mixed model < 10^–2^), with increases from baseline (semi-recumbent at 30°) to the straight 0° (*P* = 0.01) and to the (semi-recumbent at 30°) final (*P* = 0.04) positions (Table 2).

Changes in norepinephrine doses were significant (*P* for the overall effect of position in mixed model < 10^–2^), with higher doses in the straight 60° and 90° positions than at baseline (semi-recumbent at 30°) (*P* < 10^–2^ for both positions in post-hoc analysis) (Table 2).

Cardiac output varied significantly during verticalization (P for the overall effect of position in mixed model < 10^–2^), with significant decreases between baseline (semi-recumbent at 30°) and the straight 60°, 90°, and final semi-recumbent at 30° positions (*P* < 10^–2^ for all post-hoc) (Table 2). The same trend was found for stroke volume (*P* for the overall effect of position in mixed model < 10^–2^), with a significant decrease between baseline (semi-recumbent at 30°) and the straight 60° and 90° positions (*P* = 0.02 and *P* = 0.01, respectively) (Table 2). Changes in serum lactate levels during the procedure were significant (*P* for the overall effect of position in mixed model = 0.04) yet modest, with no between-position differences in post-hoc analysis (Table 2). Two patients presented hemodynamic instability at the 90° position, necessitating an interruption of the verticalization procedure and return to previous step; in both cases, norepinephrine doses were above 1 μg kg^−1^ min^−1^ at baseline. Other hemodynamic variables are reported in Table 2.

#### Gas exchanges and pulmonary shunt fraction

During verticalization, PaO_2_/FiO_2_ and PaCO_2_ changed significantly (P for overall effects of position in mixed model < 10^–2^ for both variables), with significant increases in both parameters between baseline (semi-recumbent at 30°) and the straight 60° and 90° positions (*P* = 0.03 for both comparisons for PaO_2_/FiO_2_; *P* = 0.04 and *P* < 10^–2^, respectively, for PaCO_2_ (Table 2).

The pulmonary shunt fraction was modified by the procedure (*P* for the overall effect of position in mixed model < 10^–2^, with significant decreases between baseline (semi-recumbent at 30°) and the straight 30°, 60°, 90°, and final semi-recumbent at 30° positions (*P* = 0.01, *P* < 10^–2^, *P* < 10^–2^, and *P* = 0.02, respectively) (Fig. 2 and Table 2).

### Safety endpoints

There were no technical issues regarding the bed or associated equipment during the trial. During the verticalization procedure, two patients required study interruption (as anticipated by study protocol) because of decrease in mean arterial pressure and sharp increase in norepinephrine doses during last step of verticalization at 90°. There were no adverse events such as unplanned extubation, endo-tracheal tube obstruction, pressure ulcers, new occurring arrythmia, or cardiac arrest.

More comprehensive analyses can be found in the supplementary section, including the P-values for comparisons between each of the positions (Supplementary Material 1: Appendix File 5, 6, 7 and 8).

## Discussion

In this study, verticalizing the bed from a flat 0° position to a fully vertical 90° position resulted in several notable respiratory and hemodynamic effects. The primary findings indicated that verticalization from 0° to 60° increased ΔP_L_ and EELV, decreased C_CW_ and increased both PaCO_2_ and the PaO_2_/FiO_2_ ratio. Additionally, verticalization led to decrease in cardiac output with several serious hemodynamic event. However, it is important to note that these effects did not change significantly with further verticalization beyond 60°.

This pioneering study provides new insights into the direct influence of progressive verticalization on respiratory mechanics, oxygenation, and, fundamentally, on hemodynamic changes. The results underscore the necessity of balancing improved oxygenation with potential adverse effects on respiratory mechanics and hemodynamics when determining the optimal angle of bed inclination for patients.

### Effects of verticalization on pulmonary mechanics (0 to 90°)

Studies have shown that shifting from a semi-recumbent to a supine-flat position in ARDS patients under mechanical ventilation significantly affects respiratory mechanics. Marrazzo et al. demonstrated that changing trunk inclination from 0° to 40° increased driving and transpulmonary pressure while decreasing both C_L_ and C_CW_ in two studies on 20 COVID-19-associated ARDS [1, 2]. Mezidi et al. found that moving from a supine-flat to a 30° semi-recumbent position significantly reduced E_L_ and E_CW_ in ARDS patients [20]. These results are consistent with our study, where progressive verticalization deteriorated pulmonary mechanics with or without hip flexion [1, 2, 10, 20].

At 0°, the kinetic energy from the ventilator was mainly directed to the lungs, but with significant redistribution to the chest wall during verticalization (Supplementary Material 1: Appendix Figures E7). Mechanical power increased without changes in PEEP, V_T_, respiratory rate, or resistance, correlating with E_L_/E_RS_ and E_CW_/E_RS_ ratios between 30° and 60° (Pearson coefficient = 0.5, P < 0.05). These findings suggest that verticalization increases intra-abdominal pressure, affecting ventilatory mechanics. Literature indicates that the increase in E_CW_ may result from thoracic compression due to abdominal organ displacement [6–9]. The thoracic support strap used for patient safety may also have decreased C_CW_.

PEEP may have influenced the observed changes. In our study, PEEP was set at 30° semi-seated baseline position and not modified. At 0°, lung collapse areas may appear, while with verticalization, alveolar overdistension areas may develop [20]. Verticalization combined with optimized PEEP, could mitigate these effects by operating within a more favorable range of pressure–volume curves, reducing lung stress and strain.

### Effects of verticalization on EELV (0 to 90°)

Bed verticalization from 0° to 30° and from 30° to 60° significantly improves EELV, with no further improvement from 60° to 90°. Strain decreases significantly only from 0° to 60°. This may be due to regional lung volume redistribution caused by gravity, enhancing dependent lung region expansion and alveolar recruitment. However, increased ΔPL suggests possible associated overdistension, supported by other parameters we observed, such as increase in PaCO_2_ from 30° to 60°.

These findings align with literature indicating that EELV and oxygenation improvements during trunk inclination in certain responsive ARDS patients can be accompanied by overdistension [3, 4] [5].

### Effects of verticalization on oxygenation and shunt (0 to 90°)

As in the studies by Richard et al. and Dellamonica et al., oxygenation improved significantly with verticalization [3, 4] from 0° to 30°, accompanied by a comparable improvement in pulmonary shunt for the same inclinations. Aligning with EELV increase from 0° to 30°, this may reflect alveolar recruitment combined with pulmonary blood flow redistribution during verticalization. This supports the observations by Gattinoni et al. who studied the effects of PEEP and showed that its increase improves oxygenation by reducing shunt, partly through blood flow redistribution to better-ventilated areas [21]. The reduction in pulmonary shunt can also be attributed to the decreased cardiac output observed up to 90°. Indeed, Dantzker and Lynch demonstrated that reducing cardiac output decreases pulmonary shunt by reducing perfusion in low V/Q areas, thus improving oxygenation [22, 23].

Conversely, we did not observe additional benefits in terms of oxygenation and shunt beyond 30°, suggesting diminishing or compensating effects. Beyond this angle, the increase in EELV may reflect overdistension, reducing regional pulmonary blood flow through capillary compression, and thus decreasing oxygenation efficiency. Pulmonary blood flow redistribution might lead to a V/Q mismatch if well-ventilated areas are not adequately perfused [24, 25]. Additionally, these phenomena can be exacerbated by decreased venous return and cardiac output during verticalization, which can reduce pulmonary perfusion and counteract the benefits of alveolar recruitment [26, 27].

### Effects of verticalization on PaCO2 and ventilatory efficiency (0 to 90°)

The increase in PaCO_2_ observed from 0° to 60° can be attributed to a combination of overdistension and redistribution of pulmonary blood flow, as suggested by previous observations and literature [26, 27]. Additionally, the reduction in venous return and cardiac output can decrease the alveolar perfusion, thus altering CO_2_ elimination (VCO_2_). We did not observe an increase in the V_D_/V_T_ ratio, but it is possible that the decrease in VCO_2_ and FeCO_2_ we observed, although not significant, mitigates this phenomenon. Indeed, according to the equation we used, neither the respiratory rate nor the V_T_ has varied significantly. Furthermore, we are limited to an overall analysis of ventilatory efficiency, which results from a combination of regional phenomena of alveolar recruitment and overdistension, involving the reaeration of initially poorly ventilated areas and increased trapping in already well-ventilated areas.

Finally, the estimation of V_D_/V_T_ with this formula requires homogeneous ventilation and perfusion, which is rarely the case in patients with ARDS. Additionally, variations in cardiac output, intrathoracic pressures, and pulmonary shunt can influence parameters such as PaCO_2_, VCO_2_ and FeCO_2_, making the estimation of V_D_/V_T_ less precise, as demonstrated in studies by Dianti and Beitler. [28, 29].

### Effects of verticalization on hemodynamics (0 to 90°)

Verticalization adversely affects hemodynamics, decreasing cardiac output and stroke volume at each stage. Reduction in venous return due to blood volume redistribution to lower body regions is a most apparent cause [30]. Norepinephrine increased from 30° to 90°, while MAP decreased between 0° and 60° despite following the study’s hemodynamic protocol, highlighting the potential challenges for hemodynamic management. Moreover, two patients exhibited hemodynamic instability at 90°, requiring experimental procedure interruption.

We also observed a decrease in RAP, PAP, and PAOP, along with an increase in PVR. First, the decrease in cardiac output may trigger autoregulatory mechanisms in the pulmonary circulation. The inverse correlation between the variation in CO and that of PVR that we observed from 0° to 30° (Pearson = 0.6), from 30° to 90° (Pearson = 0.8), and from 60° to 90° (Pearson = 0.6) (*P* < 0.05) (Table E7), strongly supports this hypothesis. As described by West and Dollery, the reduction in pulmonary blood flow is accompanied by an increase in PVR due to compensatory pulmonary capillary vasoconstriction, and a decrease in PAP if this mechanism is overwhelmed, especially in ARDS cases [31, 32]. Overdistension may also play a role by increasing the compression of small capillaries [33]. This idea is supported by the increase in ΔP_L_ we observed, which could explain the increase in PVR and the decrease in PAOP, as proposed by Permutt et al., with a more pronounced effect when cardiac output is reduced [34]. Verticalization may also induce a decrease in PAP through reduced venous return due to blood volume redistribution [30], which appeared as a significant decrease in RAP up to 60° in our study. Lastly, the increase in thoracic pressure could also play a role. Although it does not seem to have a direct impact on PAP in spontaneous breathing according to a physiological study on horses [35], increased pleural pressure can cause mediastinal compression in mechanical ventilation, contributing to reduced venous return [32].

Finally, it is important to consider these hypotheses carefully, as the patients were not in a normal physiological state. The interaction of numerous mechanisms, such as verticalization, hemodynamic variations, and changes in pulmonary mechanics, along with the implications of ARDS, significantly complicates the interpretation of our results.

### Limitations

First, the evaluation time per phase was relatively short, which may have limited the effects of patient positioning at each step. Second, the position sequences were not randomized, and patients were not returned to the initial position between each step, introducing a potential risk of a cumulative carryover effect, making the specific and independent effects of each position unclear. Third, the small sample size could limit the generalizability of the results. Fourth, the potential displacement of the esophageal probe during the verticalization could have led to loss of its initial calibration and measurement inaccuracies. Fifth, prior optimization of ventilation parameters and lung aeration, notably with PEEP titration following a standardized alveolar recruitment maneuver, might have minimized the current results. Finally, the majority of patients included in this study had COVID-19-associated ARDS, making it uncertain if these results are applicable to other ARDS populations.

### Strengths

First, it is the first to investigate the effects of bed verticalization up to 90°, with legs extended, in patients with ARDS. Verticalization procedures have already been proposed but were limited to the 60° seated position [3, 4] and did not include evaluation of the transpulmonary driving pressure, pulmonary artery measurements, or pulmonary shunt fraction. Second, our study confirmed the simplicity and feasibility of verticalizing ICU patients, although such current hypothesis-generating results raise potential hemodynamic concerns.

## Conclusion

In early ARDS patients, bed verticalization from 0° to 90° significantly impacts pulmonary mechanics, including increases in ΔP_L_ and mechanical power, and decreases in C_CW_. Improved EELV and oxygenation were observed up to 30°, likely due to alveolar recruitment and blood flow redistribution. However, there is likely overdistension and reduced ventilatory efficiency beyond 30°, indicated by increased PaCO_2_ and unchanged PaO_2_. Verticalization also adversely affects hemodynamics, decreasing cardiac output and stroke volume while increasing norepinephrine requirements.

This pilot study suggests that strict bed verticalization (to a 90° position) is feasible in intubated ARDS patients, offering potential benefits in alveolar recruitment. However, these benefits must be weighed against the risks of pulmonary overdistension and hemodynamic instability. These findings emphasize the importance of individualizing the optimal bed angle based on each patient's clinical situation.

## Supplementary Information

Supplementary Material 1 (DOCX 2119 kb)

## Data Availability

The datasets used and/or analysed during the current study are available from the corresponding author on reasonable request.

## Abbreviations

ARDS: Acute Respiratory Distress Syndrome
BMI: Body Mass Index
C_CW_: Chest wall compliance
CHU: Centre Hospitalier Universitaire
C_L_: Lung compliance
CO: Cardiac output
CO_2_: Carbon dioxide
COVID-19: Coronavirus disease 2019
C_RS_: Static compliance of respiratory system
E_CW_: Chest wall elastance
EDV: End-diastolic volume
EELV: End-expiratory lung volume
E_L_: Lung elastance
E_RS_: Static elastance of respiratory system
FeCO_2_: Fraction of expired carbon dioxide
FiO_2_: Fraction of inspired oxygen
ICU: Intensive Care Unit
MAP: Mean arterial pressure
PAC: Pulmonary artery catheter
PaCO_2_: Arterial partial pressure of carbon dioxide
PaO_2_: Arterial partial pressure of oxygen
PAOP: Pulmonary artery occlusion pressure
PAPm: Mean pulmonary artery pressure
PBW: Predicted body weight
PEEP: Positive end-expiratory pressure
P_es_: Esophageal pressure
P_L_: Transpulmonary pressure
PP: Prone positioning
P_plat_: End-inspiratory plateau pressure
PVR: Pulmonary vascular resistance
RAP: Right atrial pressure
SaO_2_: Arterial oxygen saturation
SvO_2_: Mixed venous blood oxygen saturation
SVR: Systemic vascular resistance
V_D_: Dead-space
V_D_/V_T_: Dead-space fraction
V_T_: Tidal volume
VCO_2_: Volume of carbon dioxide eliminated per minute
V_T_CO_2_: Volume of carbon dioxide eliminated per breath
ΔP: Driving pressure
ΔP_L_: Transpulmonary driving pressure
