# Supplementary material for: Physiological effects and safety of bed verticalization in patients with acute respiratory distress syndrome
Source: Crit Care. 2024 Aug 5;28:262. doi: 10.1186/s13054-024-05013-y (PMC11299299; doi:10.1186/s13054-024-05013-y)
Supplement: Supplementary file 1 — Supplementary Material 1 (DOCX 2119 kb) [file 13054_2024_5013_MOESM1_ESM.docx]

**Appendix File 1. EXTENDED MATERIALS AND METHODS SECTION**

**Study design**

The ERECTION study was a prospective, open label, pilot physiological study in which each patient was their own control. It was conducted in two intensive care units at CHU Clermont-Ferrand (France). The trial was approved by the French ethics committee (*Comité de Protection des Personnes Ouest IV* *Nantes*) on July 2, 2019, and the European medicine agency (EudraCT 2019-A01006-51). The protocol was registered on ClinicalTrial.gov (NCT04371016) and the study was performed in accordance with the Template for Intervention Description and Replication (TIDieR) checklist. Written informed consent was obtained from a relative of each patient, or through an emergency consent procedure, with a subsequent search for consent to continue to participate. The authors vouch for the accuracy and completeness of the data, and for the fidelity of the trial to the protocol.

**Study patients**

Adult patients (≥ 18 years old) admitted to participating ICUs with early moderate to severe ARDS (< 12 h and partial pressure of arterial oxygen-to-fraction of inspired oxygen ratio [PaO_2_/FiO_2_] < 200 mmHg) according to the Berlin criteria (1) and receiving invasive controlled mechanical ventilation through an endotracheal tube or a tracheostomy, deep sedation (as reflected by a bispectral index [BIS^TM^, Medtronic, Ireland] value of 30–50), and neuromuscular blockade (to avoid asynchrony, with a train-of-four target of <2/4 at the orbicular site) were eligible. Obese (body mass index [BMI] ≥35 kg.m^-2^) and hemodynamically unstable patients (as defined by an increase of >20% in catecholamine requirements in the last hour, despite blood volume optimization to reach a mean arterial pressure target of 65–75 mmHg), were not included.

**Patient installation**

A “verticalization team” (composed of an intensive care physician, an intensive care resident, and a nurse) was called each time a patient was deemed eligible by the treating physician. After eligibility and consent were checked, patients were placed on a dedicated bed (Total Lift Bed^TM^, VitalGo Systems Inc., Arjo AB, USA) and secured with three large flexible straps at the knee, pelvis, and medio-thoracic levels. A pulmonary artery catheter (PAC) (Swan-Ganz CCOmbo V, Edwards Lifescience corporation, USA) was inserted, if not already present, for continuous hemodynamic monitoring. Patients were ventilated using an ICU ventilator allowing EELV measurement by the nitrogen washin/washout method (Engström Carestation^TM^ or Carescape R860^TM^, General Electric Healthcare, USA) (2). Tidal volume was set at 6 mL.kg^-1^ of predicted body weight (PBW). Positive end-expiratory pressure (PEEP) was titrated after the alveolar recruitment maneuver, with decremental steps, to minimize driving pressure (ΔP = P_plat_-PEEP). An esophageal balloon catheter (Nutrivent^TM^, Sidam, Italy) was inserted at a distance of 35–45 cm from the nostrils and inflated with 4 mL of air, as recommended by the manufacturer, and correct position was confirmed using the Baidur method (3). After securing the patient, the bed could be gradually verticalized from 0° to 90° without body flexion, ensuring a standing upright position (**Figure 1**).

**Positioning**

All patients started the sequence in a 30° semi-seated position (baseline). After 30 minutes, 30-minute-long successive steps at 0°, 30°, 60°, and 90° were performed, allowing progressive bed verticalization without body flexion (strict supine position on the bed). Then, patients were returned to the 30° semi-seated position for 30 minutes (**Figure 1**).

To limit the risk of low blood pressure during verticalization, all patients were continuously monitored using PAC. Fluid optimization was managed based on preload dependency data, defined as an increase of more than 10% in stroke volume after the administration of 250 mL of crystalloids, as recommended by the French Society for Anesthesia and Intensive Care Medicine (SFAR) (4). In a case of hemodynamic instability (defined as an increase of more than 20% in catecholamines doses, despite fluid optimization), the patient was positioned back to the level in the previous step.

**Measurements**

At the end of each 30-minute step, identical complete sets of data were collected, including hemodynamic data with PAC, ventilatory parameters, EELV, esophageal pressures, and arterial blood gases parameters.

Adverse events possibly related to verticalization such as patient fall from the bed, strap-induced skin lesions, accidental removal of catheters, pressure sores, tracheal tube displacement or obstruction, hemodynamic instability (defined as an increase of more than 20% in catecholamine doses, despite fluid optimization), arrhythmia, or cardiac arrest were recorded.

Hemodynamic evaluation was conducted using an arterial line (mean arterial pressure) and a PAC, with continuous measurements of cardiac output, right atrial pressure, end-diastolic volume, mean pulmonary artery pressure, systemic vascular resistance, oxygen saturation of mixed venous blood, and punctual measurements of pulmonary artery occlusion pressure and pulmonary vascular resistance.

Pulmonary mechanics were assessed by collecting or measuring the level of total PEEP (measured during an end-expiratory pause of 5 seconds), expiratory tidal volume (V_T_), respiratory rate (RR), plateau pressure (P_plat_, measured during an end-inspiratory pause of 3 seconds), end-inspiratory and end-expiratory esophageal pressure (P_es_), and EELV. The estimated pulmonary dead-space fraction (V_D_/V_T_) was calculated using a rearranged alveolar equation for PaCO_2_ with measured volume of CO_2_ eliminated per minute (VCO_2_) (5, 6).

Arterial blood was sampled to assess PaO_2_/FiO_2_, PaCO_2_, arterial pH, serum bicarbonate, and venous blood to evaluate the pulmonary shunt fraction using the Berggren equation (7).

**Calculations**

Driving pressure (ΔP) was computed as P_plat_ – total PEEP. Static compliance of the respiratory system (C_RS_) was computed as V_T_/ΔP. Transpulmonary driving pressure (ΔP_L_) was computed as ΔP-ΔP_es_, where ΔP_es_ was the difference between end-inspiratory and end-expiratory P_es_. Chest wall compliance (C_CW_) was computed as V_T_/ΔP_es_ and lung compliance (C_L_) as V_T_/ΔP_L_. Strain was computed as V_T_/EELV (8). Mechanical power was computed as RR.{V_T_^2^ .[1/2.EL_RS_+RR.(1+I:E)/(60.I:E).R_aw_]+V_T_.PEEP}, where EL_RS_ was the elastance of the respiratory system, I:E the inspiratory-to-expiratory time ratio, and R_aw_ the airway resistance (9). The estimated V_D_/V_T_ was calculated using a rearranged alveolar equation for PaCO_2_, as 1-([0.86.VCO_2_]/[V_T_.RR.PaCO_2_]) (10). The CO_2_ eliminated per breath (V_T_CO_2_) was calculated as VCO_2_/RR. The fraction of expired CO_2_ (FeCO_2_) was calculated as V_T_CO_2_/V_T_.

Statistical analysis

The aim of this protocol is to study the influence of bed verticalization on transpulmonary driving pressure in patients presenting with moderate to severe ARDS under invasive mechanical ventilation. To date, no data concerning inter- and intra-subject variability are referenced in the literature for the populations studied and present study might be seen as a pilot study. Thus, the justification for the size of the study is based mainly on recruitment capacity, the time required for experimentation, the experience of the team involved in the research and simulations concerning the effect size with regard to the recommendations concerning pilot feasibility studies (11).

According to this recommendation: ”Most methodological papers that focus on recommendations about sample size requirements for pilot trials assume that the main aim of such a trial is to estimate a quantitative measure such as the variance (or standard deviation) of an effect size to inform the sample size calculation for a future definitive RCT. Methods focus on the precision with which such estimates can be obtained. There are several relevant papers (12–14). Among these, Whitehead et al. suggests that the size of a pilot trial should be related to the size of the future definitive RCT (15). For such a trial designed with 90% power and two sided 5% significance, they recommend pilot trial sample sizes for each treatment arm of 75, 25, 15, and 10 for standardised effect sizes that are extra small (0.1), small (0.2), medium (0.5), or large (0.8), respectively.” and works from Schramm et al. (Actu Anaesthesiol Scund, 1997, Neurosurgical anesthesia, 1998), Shaheen et al. (Neurology India 2000) and Cooper et al. (NEJM 2011).

Approximate quantification of the prospective effect size can be achieved by literature search, expert knowledge or the use of pilot studies. It is also possible to explore different scenarios, for want of a better term, as proposed by Cohen (1988), using as a starting point conventional effect sizes which for Student's test are: small (ES = 0.2), medium (ES = 0.5) and large (ES = 0.8). The larger the effect size, the greater the deviation from the null hypothesis, and conversely, the null hypothesis corresponding to the nullity of the effect size (ES = 0). In view of the above, we plan to include 30 patients, which should enable us to demonstrate an effect size of around 0.8 for a two-sided first species risk of error of 2.5% (correction due to multiple comparisons), a power of over 80% and an intra-individual correlation coefficient of 0.5 (correlation due to the several positions evaluated for the same patient).

Categorical data were expressed as numbers and percentages, and continuous data as median and interquartile range [IQR]. The normality was studied by the Shapiro–Wilk test and the homoscedasticity by the Fisher–Snedecor test. All results were compared according to the positions by mixed effects models, with time and patient’s position as variables. A two-sided p-value of less than 0.05 was considered to indicate statistical significance. All analyses were performed using Prism 8 (GraphPad Software, La Jolla, CA, USA) and Stata version 14 (StataCorp, College Station, TX, USA).

**Appendix File 2. NITROGEN WASHOUT/WASHIN TECHNIQUE**

Briefly, using FRC INview module of the ventilator (Engström Carestation or Carescape^TM^ R860, General Electrics, Healthcare, USA) accepted O_2_ level change to perform nitrogen washout calculation was set at 10% downwards. A single measurement was performed at the end of each 30 minutes positioning steps. Functional Residual Capacity (FRC) calculation were performed over 20 breaths. Technic was first described in 1903 by Durig (16) and developed by Fretschner at al. in 1993 (17) but was limited in daily use because of large FiO_2_ change requested (30%), and the synchronisation between gas measurement and flow measurement. Olegard et al. in 2005 developped a novel method only based on oxygen and carbon dioxide measurements (18). FiO_2_ changes were limited to 10% and has been validated against helium dilution and CT-scan evaluation of EELV in *Critical Care* in 2008 by Chiumello et al (2).

The nitrogen washout/washin technique is based on the following principle: the gas lung volume, at baseline, includes a volume of nitrogen (V_(1)_N_2_) that is determined by the alveolar fraction of nitrogen (F_A_N_2(1)_) (which varies inversely to the alveolar oxygen fraction) and by the EELV accordingly to the following relation:

V_(1)_N_2_ = F_A_N_2(1)_ × EELV (a)

If the alveolar nitrogen fraction (F_A_N_2(2)_) is changed by changing the FiO_2_, a new nitrogen volume (V_(2)_N_2_) will be present in the lung after the equilibrium time:

V_(2)_N_2_ = F_A_N_2(2)_ × EELV (b)

Assuming that after changing the FiO_2_ the total EELV does not change until the new equilibrium in alveolar gas composition is reached, by subtracting term by term in the equation (a) and (b) the following relation holds true:

VN_2(1)_ - VN_2(2)_ = (F_A_N_2(2)_ - F_A_N_2(1)_) × EELV

as the changes in FAN_2_ are specular to the changes in FiO_2_, i.e. ΔFAN_2_ = -(FiO_2(1)_ - FiO_2(2)_), the EELV can be calculated as:

EELV = ΔN_2_ (ml)/ΔFiO_2_

where ΔN_2_ equals the nitrogen exhaled after the change of inspired FiO_2_ until the equilibration time is reached (about 20 breaths).

The algorithm of the nitrogen washout/washin technique employed by the Engström Carestation is detailed by Olegard and colleagues (18). Nitrogen concentration in expired and inspired air is not directly measured but estimated from the end tidal concentrations of oxygen and carbon dioxide:

ETN_2_ (mmHg) = 713 - ETCO_2_ (mmHg) - ETO_2_ (mmHg)

The alveolar ventilation was calculated as:

Alveolar tidal volume expired = VCO_2_/ETCO_2_ × RR

Alveolar tidal volume inspired = Alveolar tidal volume inspired + ((VCO_2_/RQ + VCO_2_)/RR)

Inspired and expired nitrogen volumes were calculated as:

Expired nitrogen volume = ETN_2_/713 × Alveolar tidal volume expired

Inspired tidal volume = Inspired nitrogen fraction × alveolar tidal volume inspired.

**Appendix File 3. PULMONARY ARTERY CATHETER**

**Introduction and placement**

Introduction was exclusively performed through a 8.5Fr introducer (Arrow) in right jugular internal vein under ultrasound guidance. PAC (CCOmbo V, 7.5 Fr, model 774F75; Edwards Lifesciences, Irvine, CA, USA) was advanced after distal lumen being attached to a pressure transducer. Correct placement was obtained after identification of wedge position (approximately at 50 cm). Systematic chest X-ray was performed before any measurements to ensure that PAC is in pulmonary artery (mostly right).

Zeroing was performed by opening the stopcock to atmospheric pressure and calibrating monitor (Vigilance II, Edwards Lifesciences, Irvine, CA, USA).

Levelling was performed on the phlebostatic axis, usually above 5 cm below the sternal angle in supine position. While performing stepwise verticalization, other reference for positioning should be adopted, as described by Song et al. in 2017. For CVP monitoring, the reference transducer was placed at an intersection of a transverse plane cutting the 4th sternochondral joint and a perpendicular line crossing four-fifths of the anteroposterior diameter of thorax from the back. For PAOP monitoring, the reference transducer was placed at an intersection of the same plane cutting the 4th sternochondral joint and a perpendicular line crossing three-fifths of the AP diameter of thorax from the back. Correct placement of transducers was checked after any position change and before any measurement.

Blood samples were obtained from distal lumen to measure SvO_2_, in dedicated heparinized syringes (BD Diagnostics, Le Pont de Claix, France). Blood samples were promptly analyzed after constant homogenization in the ICU on a GEM® Premier 5000 blood gas testing system (Werfen, Bedford, MA, USA).

For optimal evaluation of shunt, concomitant arterial blood sample from radial arterial catheter was obtained and analyzed in the same condition. (19–21)

**Appendix File 4. DESCRIPTION OF ADVERSE EVENTS RELATED TO VERTICALIZATION**

Adverse events occurring during study period or after completion during ICU stay. Any adverse event will be reported as anticipated in study protocol as requested by French law. Unanticipated problems follow published definitions as described in Reviewing and Reporting of Unanticipated Problems Involving Risks to Subjects or Others and Adverse Events: OHRP Guidance (2007) (22).

| Patient fall from the bed | Verticalization team will firstly call for additional help. Bed will be placed back to supine position and patient re-installed on bed. Other adverse events will be managed as described in other cases. |
| --- | --- |
| Strap induced skin lesions | Prevention of skin lesions are firstly limited by dedicated straps designed by manufacturer to limit skin damage. Secondly, before any bed verticalization and new step, contacts between skin and straps will be controlled. Additional dressings such as soft dedicated cushion an/or dressing will be applied in case of contacts considered as potentially damaging skin.  In case of skin lesion occurrence despite prevention measures, management will be left to the discretion of clinicians in charge of patient, following recommendations on management of skin lesions depending of characteristics. |
| Accidental catheter or equipment removal | Verticalization team will firstly call for additional help. Bed will be placed back to supine position. Any accidental catheter of equipment removal will be managed as requested by clinical condition following recommendations and clinical habits of ICU teams. |
| Pressure sores | Pressure sores will be managed in accordance with skin lesions specialists available at inclusion sites, and following of available recommendations will be encouraged. |
| Tracheal tube displacement or obstruction | Verticalization team will firstly call for additional help. Bed will be placed back to supine position. Tracheal tube displacement or obstruction will be managed as requested by clinical condition following recommendations and clinical habits of ICU teams. |
| Hemodynamic instability as defined as an increase of more than 20% in catecholamine doses despite fluid optimization | Verticalization team will firstly call for additional help. Bed will be placed back to previous hemodynamically stable bed position. Assessment of any additional fluid requirement will be done following indications of PAC. Fluid test loading with 250 mL of crystalloids will be monitored on stroke volume as recommended. Further additional fluids will be given in case of rise of stroke volume of more than 20%. |
| New occurring arrythmia | Verticalization team will firstly call for additional help. Bed will be placed back to supine position. Newly occurring arrythmia will be managed according to hemodynamic tolerance following guidelines (23, 24) and good clinical practices. |
| Cardiac arrest | Verticalization team will firstly call for additional help. Bed will be placed back to flat supine position. Cardiac arrest management will follow guidelines (25) |

**Appendix File 5. FIGURE OF ESOPHAGEAL PRESSURE VARIATIONS DURING VERTICALIZATION**


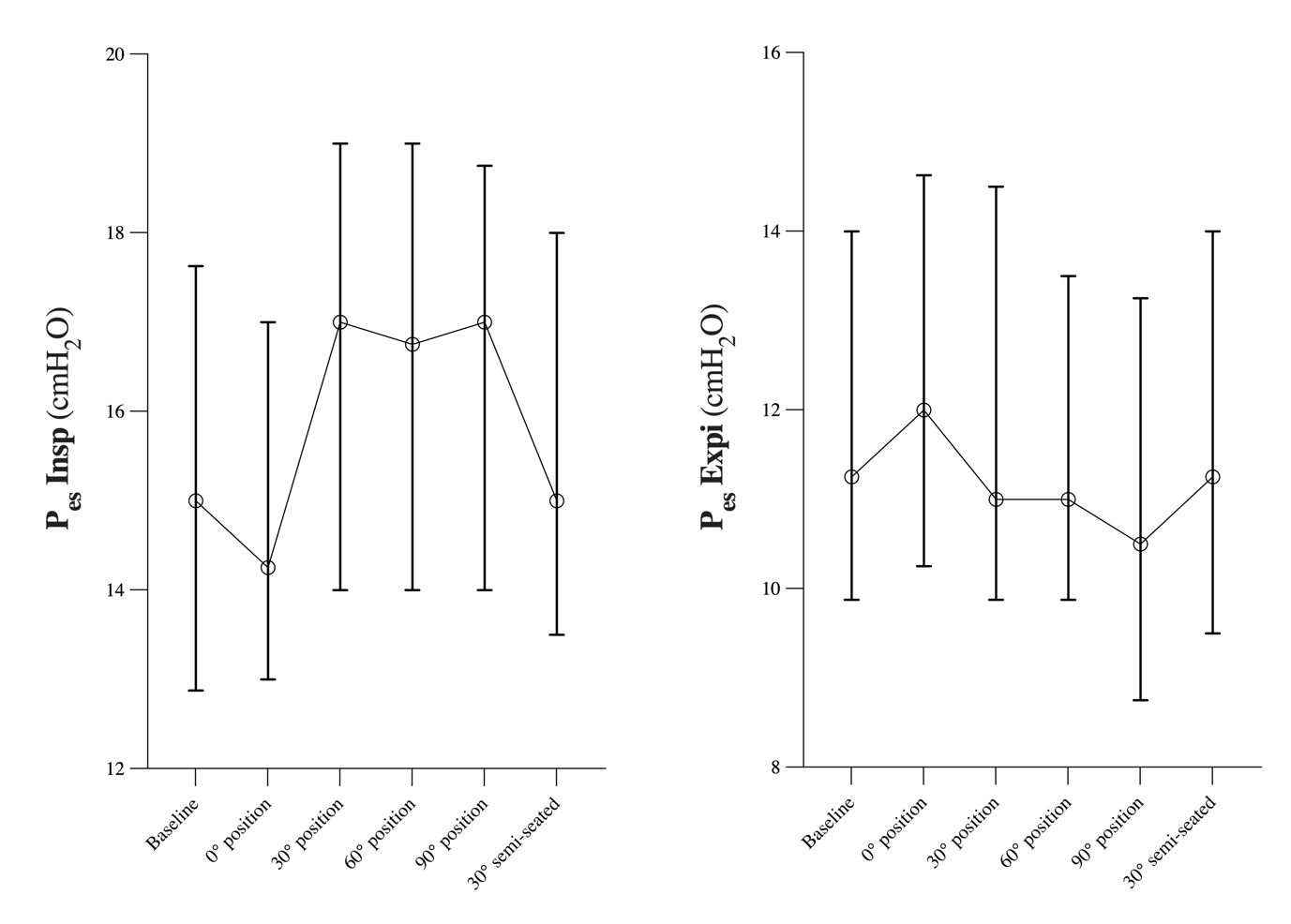


**Figure E1.** **Esophageal pressure values evaluated at each position, from baseline (30° semi-seated position) to the standing upright position (90°), and a repositioning to 30° semi-seated.** Values are reported as column with connected median and interquartile range. We observed a moderate decrease in expiratory esophageal pressure (P_es_Expi ) and an increase in inspiratory esophageal pressure (P_es_Insp) during verticalization. These variations could partly be explained by the displacement of the mediastinum structures and the redistribution of tidal volume within the thorax, but additional data would be needed to confirm this, such as computed tomography scans with volumetric analysis at each mobilization step, which is challenging to implement. However, at each step, we used the Baydur method to confirm the correct position of the esophageal catheter, which is essential for obtaining accurate measurements of pleural pressure.

**Appendix File 6. TABLES OF COMPARISONS OF THE DIFFERENT POSITIONS WHICH EACH OTHER.**

| P-value for multiple comparisons of position *vs* A | A *vs* B | A *vs* C | A *vs* D | | A *vs* E | | A *vs* F | |  |
| --- | --- | --- | --- | --- | --- | --- | --- | --- | --- |
| MAP, mmHg | **0.01*** | >0.99 | 0.95 | | >0.99 | | **0.01*** | |  |
| Heart rate, min^-1^ | 0.75 | 1.00 | **<0.01***** | | **<0.01****** | | **0.01*** | |  |
| Stroke volume, mL | 0.32 | 0.97 | **0.02*** | | **0.01*** | | 0.86 | |  |
| EDV, mL | >0.99 | 1.00 | 0.88 | | **<0.01**** | | 0.26 | |  |
| CO, mL.min^-1^ | 0.7 | 0.27 | **<0.01****** | | **<0.01****** | | **<0.01***** | |  |
| RAP, mmHg | 0.31 | **<0.01****** | **<0.01****** | | **<0.01****** | | >0.99 | |  |
| SVR, mmHg.min^-1^.mL^-1^ | 0.89 | 0.45 | **<0.01****** | | **<0.01****** | | **<0.01**** | |  |
| SvO_2_, % | 0.43 | 0.93 | 0.22 | | 0.27 | | 0.23 | |  |
| PAPm, mmHg | 0.84 | **0.03*** | **0.04*** | | 0.05 | | >0.99 | |  |
| PVR, mmHg.min^-1^.mL^-1^ | 0.98 | 0.43 | **<0.01****** | | **<0.01****** | | 0.65 | |  |
| PAOP, mmHg | **<0.01**** | **<0.01**** | **<0.01****** | | **<0.01****** | | 0.96 | |  |
| Serum lactate, mmol.L^-1^ | 0.95 | 0.99 | 0.36 | | 0.17 | | 0.93 | |  |
| Norepinephrine, µg.kg^-1^.min^-1^ | 0.98 | 0.35 | **<0.01**** | | **<0.01***** | | 0.68 | |  |
| PEEP, cmH_2_O | 0.91 | 1 | 0.91 | | 0.91 | | 0.91 | |  |
| Tidal volume, mL.kg^-1^ PBW | 0.11 | **0.01*** | 0.1 | | 0.09 | | 0.99 | |  |
| Respiratory rate, min^-1^ | 1.00 | 1 | 0.62 | | 0.44 | | 0.81 | |  |
| P_plat_, cmH_2_O | 0.31 | **<0.01***** | **<0.01****** | | **<0.01****** | | 0.35 | |  |
| C_RS_, mL.cmH_2_O^-1^ | 0.87 | **<0.01**** | **<0.01****** | | **<0.01****** | | 0.99 | |  |
| C_L_, mL.cmH_2_O^-1^ | 1 | 1 | 0.8 | | 0.75 | | 0.89 | |  |
| C_CW_, mL.cmH_2_O^-1^ | 0.9 | **<0.01**** | **<0.01**** | | **<0.01***** | | 0.42 | |  |
| Driving pressure, cmH_2_O | 0.37 | **<0.01***** | **<0.01****** | | **<0.01****** | | 0.41 | |  |
| ΔP_L_, cmH_2_O | 0.99 | 0.51 | **0.01*** | | **<0.01**** | | **0.03*** | |  |
| Mechanical power, J.min^-1^ | 0.85 | >0.99 | 0.67 | | 0.97 | | 0.99 | |  |
| EELV_PBW_, mL.kg^-1^ | 1.00 | **<0.01****** | **<0.01****** | | **<0.01****** | | 0.31 | |  |
| V_D_/V_T_, % | 0.99 | 0.22 | 0.22 | | **0.04*** | | 0.97 | |  |
| Strain | 0.07 | **<0.01**** | **<0.01**** | | **<0.01**** | | 0.19 | |  |
| PaO_2_, mmHg | 0.05 | 0.32 | 0.09 | | 0.07 | | 0.89 | |  |
| PaO_2_/FiO_2_, mmHg | **0.05*** | 0.11 | **0.03*** | | **0.03*** | | 0.72 | |  |
| PaCO_2_ mmHg | 0.55 | 0.97 | **0.04*** | | **<0.01**** | | 0.69 | |  |
| FiO_2_, % | 1 | 0.93 | 0.61 | | 0.56 | | >0.99 | |  |
| FeCO_2_, % | 0.96 | 0.77 | | 0.99 | | 0.48 | | **0**.96 | |
| VCO_2_, mL.min^-1^ | 0.99 | 0.97 | | 0.99 | | 0.53 | | 0.54 | |
| Pulmonary shunt, % | 0.05 | **<0.01**** | **<0.01***** | | **<0.01**** | | **0.02*** | |  |
| Arterial pH | 0.33 | 0.33 | **<0.01****** | | **<0.01****** | | **<0.01****** | |  |
| Serum bicarbonate, mmol.L | >0.99 | 0.97 | 0.16 | | **<0.01***** | | 0.7 | |  |

**Table E1. Multiple comparisons of A *vs* other positions.** *A: Baseline position. B: 0° position. C: 30°position. D: 60° position. E: 90° position. F 30° semi-seated position.*

| P-value for multiple comparisons of position *vs* B | B *vs* A | B *vs* C | B *vs* D | B *vs* E | B *vs* F |
| --- | --- | --- | --- | --- | --- |
| MAP, mmHg | **0.01*** | 0.2 | **0.05*** | 0.43 | 0.91 |
| Heart rate, min^-1^ | 0.75 | 0.67 | **<0.01***** | **<0.01****** | **0.01*** |
| Stroke volume, mL | 0.32 | 1 | **<0.01****** | **<0.01****** | 0.23 |
| EDV, mL | >0.99 | 1 | 0.81 | 0.3 | 0.4 |
| CO, mL.min^-1^ | 0.7 | **0.03*** | **<0.01****** | **<0.01****** | **<0.01***** |
| RAP, mmHg | 0.31 | **<0.01****** | **<0.01****** | **<0.01****** | 0.66 |
| SVR, mmHg.min^-1^.mL^-1^ | 0.89 | 0.74 | **<0.01****** | **<0.01****** | **<0.01***** |
| SvO_2_, % | 0.43 | 0.06 | 0.94 | 0.8 | **<0.01**** |
| PAPm, mmHg | 0.84 | **<0.01**** | **<0.01**** | **<0.01**** | 0.96 |
| PVR, mmHg.min^-1^.mL^-1^ | 0.98 | 0.26 | **<0.01****** | **<0.01****** | 0.53 |
| PAOP, mmHg | **<0.01**** | **<0.01****** | **<0.01****** | **<0.01****** | 0.96 |
| Serum lactate, mmol.L^-1^ | 0.95 | 0.62 | 0.15 | 0.12 | 0.75 |
| Norepinephrine, µg.kg^-1^.min^-1^ | 0.98 | 0.19 | **<0.01**** | **<0.01***** | 0.25 |
| PEEP, cmH_2_O | 0.91 | 0.91 |  |  |  |
| Tidal volume, mL.kg^-1^ PBW | 0.11 | 0.35 | 0.99 | >0.99 | 1 |
| Respiratory rate, min^-1^ | 1.00 | 0.99 | 0.57 | 0.51 | 0.81 |
| P_plat_, cmH_2_O | 0.31 | **<0.01***** | **<0.01****** | **<0.01****** | 1 |
| C_RS_, mL.cmH_2_O^-1^ | 0.87 | **<0.01**** | **<0.01****** | **<0.01****** | 0.96 |
| C_L_, mL.cmH_2_O^-1^ | 1 | >0.99 | 0.93 | 0.9 | 0.66 |
| C_CW_, mL.cmH_2_O^-1^ | 0.9 | **<0.01***** | **<0.01***** | **<0.01****** | 0.11 |
| Driving pressure, cmH_2_O | 0.37 | **<0.01***** | **<0.01****** | **<0.01****** | 1 |
| ΔP_L_, cmH_2_O | 0.99 | 0.55 | **0.03*** | **0.01*** | 0.18 |
| Mechanical power, J.min^-1^ | 0.85 | 0.17 | **<0.01**** | 0.28 | 0.94 |
| EELV_PBW_, mL.kg^-1^ | 1.00 | **<0.01****** | **<0.01****** | **<0.01****** | **0.01*** |
| V_D_/V_T_, % | 0.99 | 0.86 | 0.81 | 0.63 | 0.99 |
| Strain | 0.07 | **<0.01**** | **<0.01**** | **<0.01**** | **0.04*** |
| PaO_2_, mmHg | 0.05 | **0.04*** | **<0.01**** | **<0.01**** | 0.14 |
| PaO_2_/FiO_2_, mmHg | **0.05*** | **<0.01**** | **<0.01**** | **<0.01**** | 0.12 |
| PaCO_2_ mmHg | 0.55 | 0.34 | **0.01*** | **0.01*** | 0.29 |
| FiO_2_, % | 1 | 0.89 | 0.72 | 0.66 | >0.99 |
| FeCO_2_, % | 0.96 | 0.58 | 0.86 | 0.36 | **0**.99 |
| VCO_2_, mL.min^-1^ | 0.99 | 0.58 | 0.99 | 0.41 | 0.35 |
| Pulmonary shunt, % | 0.05 | **<0.01****** | **<0.01****** | **<0.01****** | **<0.01****** |
| Arterial pH | 0.33 | **0.01*** | **<0.01****** | **<0.01****** | **<0.01****** |
| Serum bicarbonate, mmol.L | >0.99 | 0.96 | **0.01*** | **<0.01****** | 0.72 |

**Table E2. Multiple comparisons of B *vs* other positions.** *A: Baseline position. B: 0° position. C: 30°position. D: 60° position. E: 90° position. F 30° semi-seated position.*

| P-value for multiple comparisons of position *vs* C | C *vs* A | C *vs* B | C *vs* D | C *vs* E | C *vs* F |
| --- | --- | --- | --- | --- | --- |
| MAP, mmHg | >0.99 | 0.2 | 0.97 | >0.99 | 0.12 |
| Heart rate, min^-1^ | 1.00 | 0.67 | **<0.01**** | **<0.01****** | **0.01*** |
| Stroke volume, mL | 0.97 | 1 | **0.02*** | **<0.01**** | 0.42 |
| EDV, mL | 1.00 | 1 | 0.72 | 0.17 | 0.07 |
| CO, mL.min^-1^ | 0.27 | **0.03*** | **<0.01**** | **<0.01****** | 0.09 |
| RAP, mmHg | **<0.01****** | **<0.01****** | **<0.01****** | **<0.01***** | **<0.01***** |
| SVR, mmHg.min^-1^.mL^-1^ | 0.45 | 0.74 | **<0.01***** | **<0.01****** | **0.01*** |
| SvO_2_, % | 0.93 | 0.06 | **0.04*** | 0.06 | 0.25 |
| PAPm, mmHg | **0.03*** | **<0.01**** | 1 | 0.96 | 0.34 |
| PVR, mmHg.min^-1^.mL^-1^ | 0.43 | 0.26 | 0.46 | 0.28 | 0.99 |
| PAOP, mmHg | **<0.01**** | **<0.01****** | **<0.01***** | **<0.01***** | 0.06 |
| Serum lactate, mmol.L^-1^ | 0.99 | 0.62 | 0.66 | 0.37 | 1 |
| Norepinephrine, µg.kg^-1^.min^-1^ | 0.35 | 0.19 | **<0.01**** | **<0.01**** | 0.99 |
| PEEP, cmH_2_O | 1 | 0.91 | 0.91 | 0.91 | 0.91 |
| Tidal volume, mL.kg^-1^ PBW | **0.01*** | 0.35 | 0.86 | 0.8 | 0.83 |
| Respiratory rate, min^-1^ | 1 | 0.99 | 0.7 | 0.63 | 0.9 |
| P_plat_, cmH_2_O | **<0.01***** | **<0.01***** | **<0.01****** | **<0.01****** | **<0.01***** |
| C_RS_, mL.cmH_2_O^-1^ | **<0.01**** | **<0.01**** | **<0.01***** | **<0.01***** | **<0.01**** |
| C_L_, mL.cmH_2_O^-1^ | 1 | >0.99 | 0.82 | 0.73 | 0.71 |
| C_CW_, mL.cmH_2_O^-1^ | **<0.01**** | **<0.01***** | 0.31 | **0.04*** | 0.81 |
| Driving pressure, cmH_2_O | **<0.01***** | **<0.01***** | **<0.01****** | **<0.01****** | **<0.01***** |
| ΔP_L_, cmH_2_O | 0.51 | 0.55 | **0.02*** | **0.02*** | **0.01*** |
| Mechanical power, J.min^-1^ | >0.99 | 0.17 | 0.17 | 0.98 | 0.44 |
| EELV_PBW_, mL.kg^-1^ | **<0.01****** | **<0.01****** | **<0.01***** | **0.04*** | **<0.01**** |
| V_D_/V_T_, % | 0.22 | 0.86 | >0.99 | 0.98 | 0.91 |
| Strain | **<0.01**** | **<0.01**** | 0.75 | 0.15 | 0.37 |
| PaO_2_, mmHg | 0.32 | **0.04*** | >0.99 | >0.99 | 0.76 |
| PaO_2_/FiO_2_, mmHg | 0.11 | **<0.01**** | 0.8 | 0.77 | 0.5 |
| PaCO_2_ mmHg | 0.97 | 0.34 | **0.02*** | **<0.01**** | 0.85 |
| FiO_2_, % | 0.93 | 0.89 | 0.88 | 0.81 | 1 |
| FeCO_2_, % | 0.77 | 0.58 | 0.99 | 0.81 | **0**.14 |
| VCO_2_, mL.min^-1^ | 0.97 | 0.58 | 0.83 | 0.82 | 0.08 |
| Pulmonary shunt, % | **<0.01**** | **<0.01****** | 0.94 | 0.65 | 1 |
| Arterial pH | 0.33 | **0.01*** | **<0.01****** | **<0.01****** | **<0.01****** |
| Serum bicarbonate, mmol.L | 0.97 | 0.96 | 0.68 | 0.24 | 0.7 |

**Table E3. Multiple comparisons of C *vs* other positions.** *A: Baseline position. B: 0° position. C: 30°position. D: 60° position. E: 90° position. F 30° semi-seated position.*

| P-value for multiple comparisons of position *vs* D | D *vs* A | D *vs* B | D *vs* C | D *vs* E | D *vs* F |
| --- | --- | --- | --- | --- | --- |
| MAP, mmHg | 0.95 | **0.05*** | 0.97 | 0.98 | 0.09 |
| Heart rate, min^-1^ | **<0.01***** | **<0.01***** | **<0.01**** | **<0.01***** | 1 |
| Stroke volume, mL | **0.02*** | **<0.01****** | **0.02*** | 0.36 | 0.75 |
| EDV, mL | 0.88 | 0.81 | 0.72 | 0.98 | 0.99 |
| CO, mL.min^-1^ | **<0.01****** | **<0.01****** | **<0.01**** | **0.0373*** | >0.99 |
| RAP, mmHg | **<0.01****** | **<0.01****** | **<0.01****** | 0.83 | **<0.01****** |
| SVR, mmHg.min^-1^.mL^-1^ | **<0.01****** | **<0.01****** | **<0.01***** | **<0.01**** | 1 |
| SvO_2_, % | 0.22 | 0.94 | **0.04*** | 0.98 | **<0.01***** |
| PAPm, mmHg | **0.04*** | **<0.01**** | 1 | 0.99 | 0.27 |
| PVR, mmHg.min^-1^.mL^-1^ | **<0.01****** | **<0.01****** | 0.46 | 0.58 | >0.99 |
| PAOP, mmHg | **<0.01****** | **<0.01****** | **<0.01***** | 0.63 | **<0.01***** |
| Serum lactate, mmol.L^-1^ | 0.36 | 0.15 | 0.66 | 0.64 | 0.94 |
| Norepinephrine, µg.kg^-1^.min^-1^ | **<0.01**** | **<0.01**** | **<0.01**** | 0.1 | 0.06 |
| PEEP, cmH_2_O | 0.91 |  | 0.91 |  |  |
| Tidal volume, mL.kg^-1^ PBW | 0.1 | 0.99 | 0.86 | 1 | 0.97 |
| Respiratory rate, min^-1^ | 0.62 | 0.57 | 0.7 | 1 | 0.99 |
| P_plat_, cmH_2_O | **<0.01****** | **<0.01****** | **<0.01****** | 0.42 | **<0.01****** |
| C_RS_, mL.cmH_2_O^-1^ | **<0.01****** | **<0.01****** | **<0.01***** | 0.63 | **<0.01****** |
| C_L_, mL.cmH_2_O^-1^ | 0.8 | 0.93 | 0.82 | 0.98 | 0.32 |
| C_CW_, mL.cmH_2_O^-1^ | **<0.01**** | **<0.01***** | 0.31 | 0.41 | 0.11 |
| Driving pressure, cmH_2_O | **<0.01****** | **<0.01****** | **<0.01****** | 0.45 | **<0.01****** |
| ΔP_L_, cmH_2_O | **0.01*** | **0.03*** | **0.02*** | 1 | **<0.01***** |
| Mechanical power, J.min^-1^ | 0.67 | **<0.01**** | 0.17 | 0.86 | **0.02*** |
| EELV_PBW_, mL.kg^-1^ | **<0.01****** | **<0.01****** | **<0.01***** | 1 | **<0.01****** |
| V_D_/V_T_, % | 0.22 | 0.81 | >0.99 | 0.97 | 0.85 |
| Strain | **<0.01**** | **<0.01**** | 0.75 | 0.99 | 0.3 |
| PaO_2_, mmHg | 0.09 | **<0.01**** | >0.99 | >0.99 | 0.15 |
| PaO_2_/FiO_2_, mmHg | **0.03*** | **<0.01**** | 0.8 | 1 | 0.07 |
| PaCO_2_ mmHg | **0.04*** | **0.01*** | **0.02*** | 0.83 | 0.87 |
| FiO_2_, % | 0.61 | 0.72 | 0.88 | 0.95 | 0.32 |
| FeCO_2_, % | 0.99 | 0.86 | 0.99 | 0.68 | 0.91 |
| VCO_2_, mL.min^-1^ | 0.99 | 0.99 | 0.83 | 0.34 | 0.98 |
| Pulmonary shunt, % | **<0.01***** | **<0.01****** | 0.94 | 0.89 | >0.99 |
| Arterial pH | **<0.01****** | **<0.01****** | **<0.01****** | **<0.01****** | 0.82 |
| Serum bicarbonate, mmol.L | 0.16 | **0.01*** | 0.68 | **<0.01***** | 1 |

**Table E4. Multiple comparisons of D *vs* other positions.** *A: Baseline position. B: 0° position. C: 30°position. D: 60° position. E: 90° position. F 30° semi-seated position.*

| P-value for multiple comparisons of position *vs* E | E *vs* A | E *vs* B | E *vs* C | E *vs* D | E *vs* F |
| --- | --- | --- | --- | --- | --- |
| MAP, mmHg | >0.99 | 0.43 | >0.99 | 0.98 | 0.18 |
| Heart rate, min^-1^ | **<0.01****** | **<0.01****** | **<0.01****** | **<0.01***** | **<0.01**** |
| Stroke volume, mL | **0.01*** | **<0.01****** | **<0.01**** | 0.36 | **0.03*** |
| EDV, mL | **<0.01**** | 0.3 | 0.17 | 0.98 | 1 |
| CO, mL.min^-1^ | **<0.01****** | **<0.01****** | **<0.01****** | **0.0373*** | 0.12 |
| RAP, mmHg | **<0.01****** | **<0.01****** | **<0.01***** | 0.83 | **<0.01****** |
| SVR, mmHg.min^-1^.mL^-1^ | **<0.01****** | **<0.01****** | **<0.01****** | **<0.01**** | 0.28 |
| SvO_2_, % | 0.27 | 0.8 | 0.06 | 0.98 | **<0.01***** |
| PAPm, mmHg | 0.05 | **<0.01**** | 0.96 | 0.99 | 0.14 |
| PVR, mmHg.min^-1^.mL^-1^ | **<0.01****** | **<0.01****** | 0.28 | 0.58 | 0.98 |
| PAOP, mmHg | **<0.01****** | **<0.01****** | **<0.01***** | 0.63 | **<0.01****** |
| Serum lactate, mmol.L^-1^ | 0.17 | 0.12 | 0.37 | 0.64 | 0.56 |
| Norepinephrine, µg.kg^-1^.min^-1^ | **<0.01***** | **<0.01***** | **<0.01**** | 0.1 | **<0.01**** |
| PEEP, cmH_2_O | 0.91 |  | 0.91 |  |  |
| Tidal volume, mL.kg^-1^ PBW | 0.09 | >0.99 | 0.8 | 1 | 0.99 |
| Respiratory rate, min^-1^ | 0.44 | 0.51 | 0.63 | 1 | 0.81 |
| P_plat_, cmH_2_O | **<0.01****** | **<0.01****** | **<0.01****** | 0.42 | **<0.01****** |
| C_RS_, mL.cmH_2_O^-1^ | **<0.01****** | **<0.01****** | **<0.01***** | 0.63 | **<0.01****** |
| C_L_, mL.cmH_2_O^-1^ | 0.75 | 0.9 | 0.73 | 0.98 | 0.24 |
| C_CW_, mL.cmH_2_O^-1^ | **<0.01***** | **<0.01****** | **0.04*** | 0.41 | **0.02*** |
| Driving pressure, cmH_2_O | **<0.01****** | **<0.01****** | **<0.01****** | 0.45 | **<0.01****** |
| ΔP_L_, cmH_2_O | **<0.01**** | **0.01*** | **0.02*** | 1 | **<0.01****** |
| Mechanical power, J.min^-1^ | 0.97 | 0.28 | 0.98 | 0.86 | 0.54 |
| EELV_PBW_, mL.kg^-1^ | **<0.01****** | **<0.01****** | **0.04*** | 1 | **<0.01****** |
| V_D_/V_T_, % | **0.04*** | 0.63 | 0.98 | 0.97 | 0.68 |
| Strain | **<0.01**** | **<0.01**** | 0.15 | 0.99 | **<0.01**** |
| PaO_2_, mmHg | 0.07 | **<0.01**** | >0.99 | >0.99 | 0.09 |
| PaO_2_/FiO_2_, mmHg | **0.03*** | **<0.01**** | 0.77 | 1 | 0.06 |
| PaCO_2_ mmHg | **<0.01**** | **0.01*** | **<0.01**** | 0.83 | 0.67 |
| FiO_2_, % | 0.56 | 0.66 | 0.81 | 0.95 | 0.06 |
| FeCO_2_, % | 0.48 | 0.36 | 0.81 | 0.68 | **0**.34 |
| VCO_2_, mL.min^-1^ | 0.53 | 0.41 | 0.82 | 0.34 | 0.25 |
| Pulmonary shunt, % | **<0.01**** | **<0.01****** | 0.65 | 0.89 | 0.87 |
| Arterial pH | **<0.01****** | **<0.01****** | **<0.01****** | **<0.01****** | **<0.01***** |
| Serum bicarbonate, mmol.L | **<0.01***** | **<0.01****** | 0.24 | **<0.01***** | 0.94 |

**Table E5. Multiple comparisons of E *vs* other positions.** *A: Baseline position. B: 0° position. C: 30°position. D: 60° position. E: 90° position. F 30° semi-seated position.*

| P-value for multiple comparisons of position *vs* F | F *vs* A | F *vs* B | F *vs* C | F *vs* D | F *vs* E |
| --- | --- | --- | --- | --- | --- |
| MAP, mmHg | **0.01*** | 0.91 | 0.12 | 0.09 | 0.18 |
| Heart rate, min^-1^ | **0.01*** | **0.01*** | **0.01*** | 1 | **<0.01**** |
| Stroke volume, mL | 0.86 | 0.23 | 0.42 | 0.75 | **0.03*** |
| EDV, mL | 0.26 | 0.4 | 0.07 | 0.99 | 1 |
| CO, mL.min^-1^ | **<0.01***** | **<0.01***** | 0.09 | >0.99 | 0.12 |
| RAP, mmHg | >0.99 | 0.66 | **<0.01***** | **<0.01****** | **<0.01****** |
| SVR, mmHg.min^-1^.mL^-1^ | **<0.01**** | **<0.01***** | **0.01*** | 1 | 0.28 |
| SvO_2_, % | 0.23 | **<0.01**** | 0.25 | **<0.01***** | **<0.01***** |
| PAPm, mmHg | >0.99 | 0.96 | 0.34 | 0.27 | 0.14 |
| PVR, mmHg.min^-1^.mL^-1^ | 0.65 | 0.53 | 0.99 | >0.99 | 0.98 |
| PAOP, mmHg | 0.96 | 0.96 | 0.06 | **<0.01***** | **<0.01****** |
| Serum lactate, mmol.L^-1^ | 0.93 | 0.75 | 1 | 0.94 | 0.56 |
| Norepinephrine, µg.kg^-1^.min^-1^ | 0.68 | 0.25 | 0.99 | 0.06 | **<0.01**** |
| PEEP, cmH_2_O | 0.91 |  | 0.91 |  |  |
| Tidal volume, mL.kg^-1^ PBW | 0.99 | 1 | 0.83 | 0.97 | 0.99 |
| Respiratory rate, min^-1^ | 0.81 | 0.81 | 0.9 | 0.99 | 0.81 |
| P_plat_, cmH_2_O | 0.35 | 1 | **<0.01***** | **<0.01****** | **<0.01****** |
| C_RS_, mL.cmH_2_O^-1^ | 0.99 | 0.96 | **<0.01**** | **<0.01****** | **<0.01****** |
| C_L_, mL.cmH_2_O^-1^ | 0.89 | 0.66 | 0.71 | 0.32 | 0.24 |
| C_CW_, mL.cmH_2_O^-1^ | 0.42 | 0.11 | 0.81 | 0.11 | **0.02*** |
| Driving pressure, cmH_2_O | 0.41 | 1 | **<0.01***** | **<0.01****** | **<0.01****** |
| ΔP_L_, cmH_2_O | **0.03*** | 0.18 | **0.01*** | **<0.01***** | **<0.01****** |
| Mechanical power, J.min^-1^ | >0.99 | 0.94 | 0.44 | **0.02*** | 0.54 |
| EELV_PBW_, mL.kg^-1^ | 0.31 | **0.01*** | **<0.01**** | **<0.01****** | **<0.01****** |
| V_D_/V_T_, % | 0.97 | 0.99 | 0.91 | 0.85 | 0.68 |
| Strain | 0.19 | **0.04*** | 0.37 | 0.3 | **<0.01**** |
| PaO_2_, mmHg | 0.89 | 0.14 | 0.76 | 0.15 | 0.09 |
| PaO_2_/FiO_2_, mmHg | 0.72 | 0.12 | 0.5 | 0.07 | 0.06 |
| PaCO_2_ mmHg | 0.69 | 0.29 | 0.85 | 0.87 | 0.67 |
| FiO_2_, % | >0.99 | >0.99 | 1 | 0.32 | 0.06 |
| FeCO_2_, % | 0.96 | 0.99 | 0.14 | 0.91 | 0.34 |
| VCO_2_, mL.min^-1^ | 0.54 | 0.35 | 0.08 | 0.98 | 0.25 |
| Pulmonary shunt, % | **0.02*** | **<0.01****** | 1 | >0.99 | 0.87 |
| Arterial pH | **<0.01****** | **<0.01****** | **<0.01****** | 0.82 | **<0.01***** |
| Serum bicarbonate, mmol.L | 0.7 | 0.72 | 0.7 | 1 | 0.94 |

**Table E6. Multiple comparisons of F *vs* other positions.** *A: Baseline position. B: 0° position. C: 30°position. D: 60° position. E: 90° position. F 30° semi-seated position.*

| **Correlation of variations from 0° to 30°** | | | | | | | | | | | | |
| --- | --- | --- | --- | --- | --- | --- | --- | --- | --- | --- | --- | --- |
|  | C_CW_ | E_CW_/E_RS_ | MP | C_L_ | E_L_/E_RS_ | EELV | V_D_/V_T_ | PAPm | PVR | RAP | PAOP | CO |
| C_CW_ | - |  |  |  |  |  |  |  |  |  |  |  |
| E_CW_/E_RS_ | **-0.83*** | - |  |  |  |  |  |  |  |  |  |  |
| MP | -0.06 | 0.29 | - |  |  |  |  |  |  |  |  |  |
| C_L_ | **-0.46*** | **0.83*** | 0.35 | - |  |  |  |  |  |  |  |  |
| E_L_/E_RS_ | **0.69*** | **-0.94*** | -0.18 | **-0.86*** | - |  |  |  |  |  |  |  |
| EELV | 0.16 | -0.22 | -0.35 | -0.17 | 0.19 | - |  |  |  |  |  |  |
| V_D_/V_T_ | -0.34 | 0.05 | -0.16 | -0.19 | 0.02 | -0.08 | - |  |  |  |  |  |
| PAPm | 0.04 | 0.01 | -0.02 | -0.05 | -0.07 | -0.04 | 0.21 | - |  |  |  |  |
| PVR | -0.18 | 0.16 | 0.09 | -0.03 | -0.10 | -0.08 | 0.21 | 0.36 | - |  |  |  |
| RAP | -0.01 | 0.05 | **-0.38*** | 0.10 | -0.18 | 0.06 | 0.02 | 0.15 | **-0.41*** | - |  |  |
| PAOP | -0.11 | 0.17 | 0.04 | 0.17 | -0.22 | 0.22 | -0.20 | -0.05 | -0.24 | 0.29 | - |  |
| CO | 0.15 | -0.18 | 0.03 | -0.09 | 0.17 | 0.08 | -0.03 | 0.08 | **-0.59*** | 0.15 | 0.07 | - |

| **Correlation of variations from 30° to 60°** | | | | | | | | | | | | |
| --- | --- | --- | --- | --- | --- | --- | --- | --- | --- | --- | --- | --- |
|  | C_CW_ | E_CW_/E_RS_ | MP | C_L_ | E_L_/E_RS_ | EELV | V_D_/V_T_ | PAPm | PVR | RAP | PAOP | CO |
| C_CW_ | - |  |  |  |  |  |  |  |  |  |  |  |
| E_CW_/E_RS_ | **-0.91*** | - |  |  |  |  |  |  |  |  |  |  |
| MP | -0.27 | **0.46*** | - |  |  |  |  |  |  |  |  |  |
| C_L_ | **-0.68*** | **0.89*** | **0.58*** | - |  |  |  |  |  |  |  |  |
| E_L_/E_RS_ | **0.82*** | **-0.94*** | **-0.51*** | **-0.94*** | - |  |  |  |  |  |  |  |
| EELV | **0.41*** | **-0.49*** | **-0.38*** | **-0.42*** | 0.35 | - |  |  |  |  |  |  |
| V_D_/V_T_ | -0.01 | 0.01 | 0.02 | -0.03 | 0.07 | -0.19 | - |  |  |  |  |  |
| PAPm | -0.19 | 0.21 | 0.32 | 0.18 | -0.18 | 0.03 | -0.19 | - |  |  |  |  |
| PVR | 0.03 | -0.04 | 0.16 | -0.06 | 0.04 | 0.12 | -0.31 | **0.56*** | - |  |  |  |
| RAP | -0.10 | 0.07 | -0.11 | 0.01 | -0.04 | 0.09 | -0.03 | **0.57*** | 0.21 | - |  |  |
| PAOP | -0.03 | -0.02 | -0.09 | 0.02 | -0.06 | 0.10 | 0.01 | 0.15 | -0.25 | 0.35 | - |  |
| CO | -0.04 | 0.10 | -0.06 | 0.08 | -0.01 | -0.05 | 0.25 | -0.34 | **-0.75*** | -016 | -0.02 | - |

| **Correlation of variations from 60° to 90°** | | | | | | | | | | | | |
| --- | --- | --- | --- | --- | --- | --- | --- | --- | --- | --- | --- | --- |
|  | C_CW_ | E_CW_/E_RS_ | MP | C_L_ | E_L_/E_RS_ | EELV | V_D_/V_T_ | PAPm | PVR | RAP | PAOP | CO |
| C_CW_ | - |  |  |  |  |  |  |  |  |  |  |  |
| E_CW_/E_RS_ | **-0.84*** | - |  |  |  |  |  |  |  |  |  |  |
| MP | 0.27 | -0.18 | - |  |  |  |  |  |  |  |  |  |
| C_L_ | -0.32 | **0.75*** | 0.01 | - |  |  |  |  |  |  |  |  |
| E_L_/E_RS_ | **0.69*** | **-0.92*** | 0.14 | **-0.85*** | - |  |  |  |  |  |  |  |
| EELV | -0.12 | 0.12 | **-0.45*** | **-0.37*** | 0.17 | - |  |  |  |  |  |  |
| V_D_/V_T_ | -0.17 | 0.19 | -0.02 | 0.01 | -0.17 | -0.13 | - |  |  |  |  |  |
| PAPm | 0.07 | -0.29 | **-0.37*** | **-0.46*** | 0.36 | **0.42*** | -0.12 | - |  |  |  |  |
| PVR | 0.23 | **-0.48*** | -0.21 | **-0.54*** | **0.44*** | 0.17 | -0.14 | **0.56*** | - |  |  |  |
| RAP | -0.09 | 0.23 | -0.18 | 0.20 | -0.11 | 0.08 | -0.12 | 0.18 | -0.17 | - |  |  |
| PAOP | 0.01 | 0.08 | 0.04 | 0.02 | -0.02 | 0.26 | -0.18 | 0.24 | -0.17 | **0.48*** | - |  |
| CO | **-0.41*** | **0.61*** | 0.18 | **0.54*** | **-0.54*** | -0.18 | 0.32 | 0.17 | **-0.59*** | 0.34 | -0.27 | - |

**Table E7. Correlation of variations of the different variables with the verticalization between different positions, from 0° to 30°, from 30° to 60° and from 60° to 90°.** *This correlation table presents the Pearson correlation coefficients between different variables for each change in position. The values range from -1 to 1, where -1 indicates a perfect negative correlation, 0 indicates no correlation, and 1 indicates a perfect positive correlation. Values marked with a '*' have P < 0.05.*

**Appendix File 7. FIGURES OF PHYSIOLOGICAL VALUES VARIATIONS DURING VERTICALIZATION**


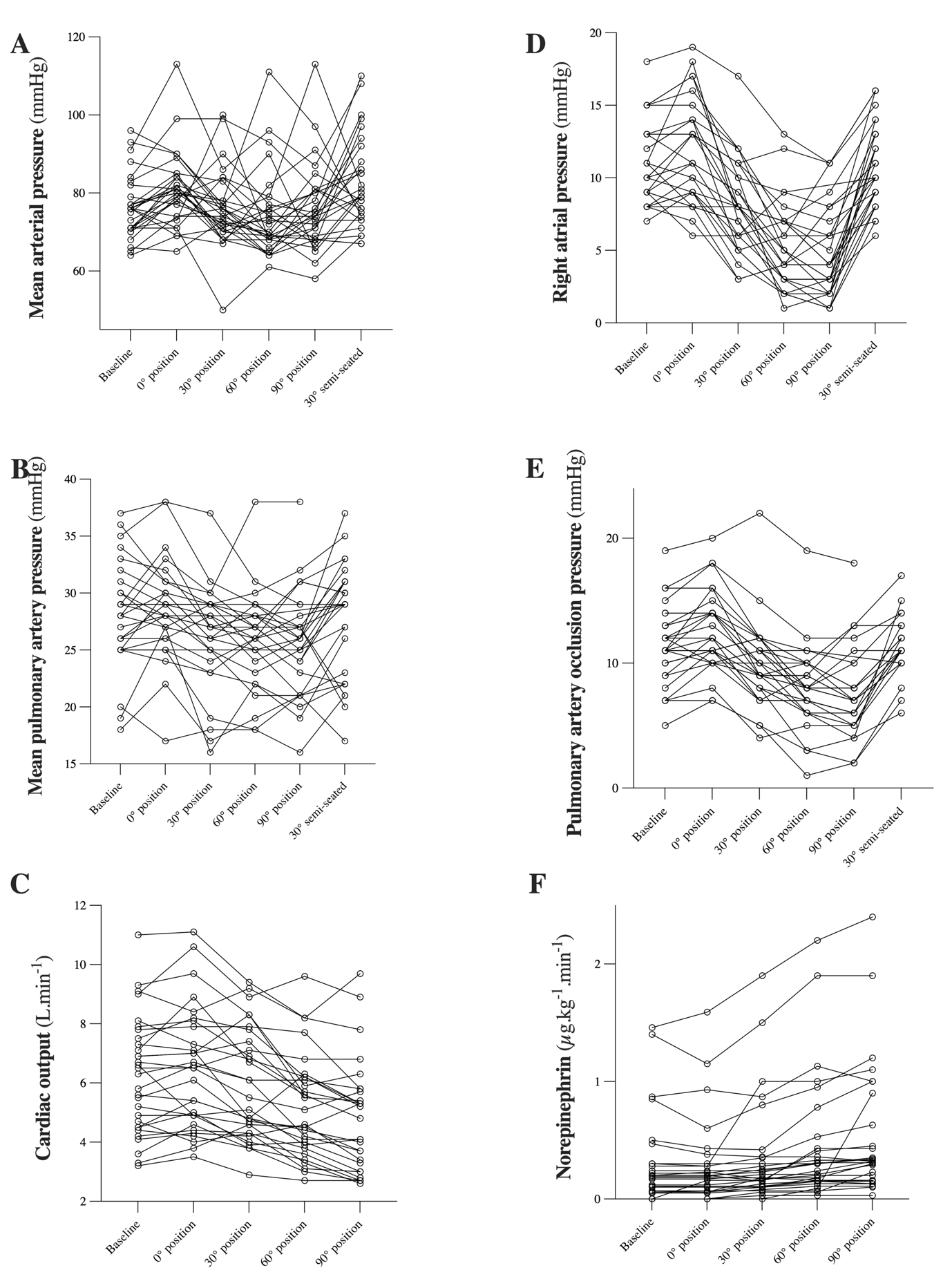


**Figure E2.** **Hemodynamic values evaluated at each position, from baseline (30° semi-seated position) to the standing upright position (90°), and a repositioning to 30° semi-seated.** Values are reported as individual values. (**A**) Mean arterial pressure. (**B**) Mean pulmonary artery pressure. (**C**) Cardiac output. (**D**) Right atrial pressure. (**E**) Pulmonary artery occlusion pressure. (**F**) Norepinephrine.

**
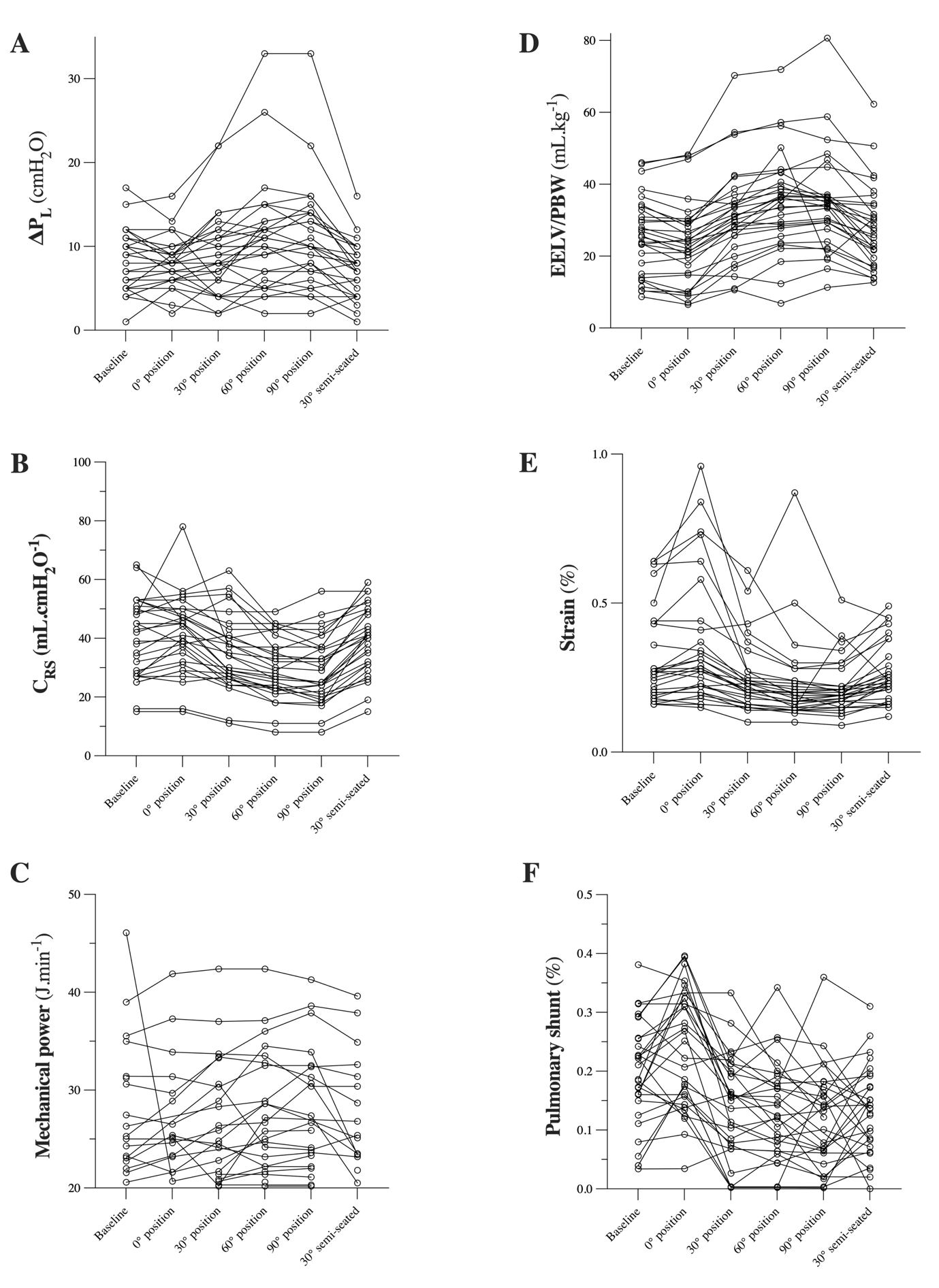
**

**Figure E3.** **Respiratory values evaluated at each position, from baseline (30° semi-seated position) to the standing upright position (90°), and a repositioning to 30° semi-seated.** Values are reported as individual values. (**A**) Transpulmonary driving pressure (ΔP_L_), computed as the difference between end-inspiratory and end-expiratory transpulmonary pressures, as measured using an esophageal balloon catheter. (**B**) Static compliance of the respiratory system (C_RS_). (**C**) Mechanical power in the different study steps. (**D**) End-expiratory lung volume, as expressed in milliliters per kilogram of predicted body weight (EELV_PBW_). (**E**) Strain, computed as the tidal volume-to-EELV ratio. (**F**) Pulmonary shunt measured using the venous-to-arterial difference in oxygen concentrations.

**Appendix File 8. FIGURES OF COMPARISON OF THE FOUR PHASES OF VERTICALIZATION WITHOUT BODY FLEXION (0° POSITION, 30° POSITION, 60° POSITION, AND 90° POSITION)**


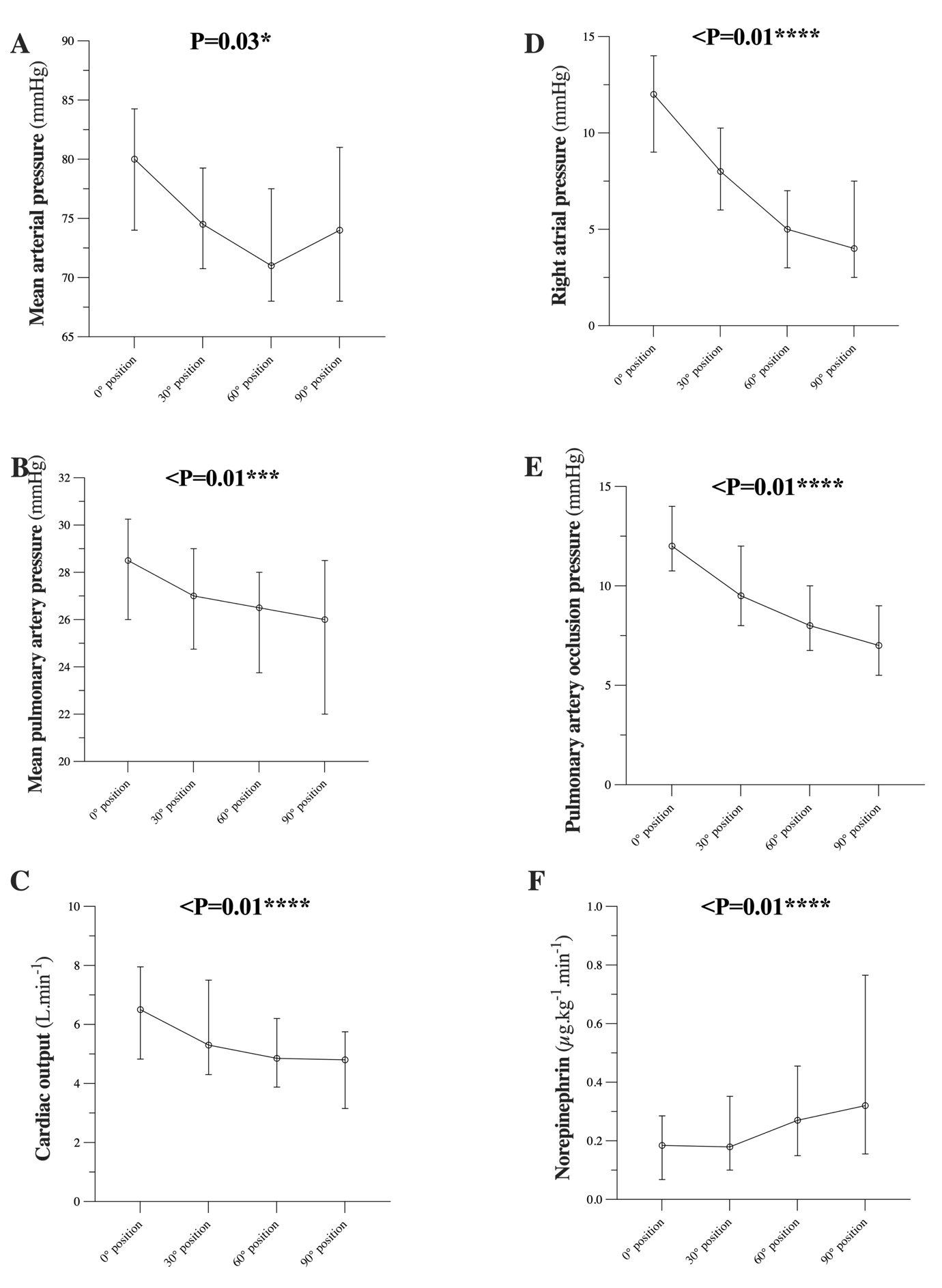


**Figure E4.** **Hemodynamic values evaluated at each position without body flexion, from 0° to 90°.** Values are reported as column with connected median and interquartile range. (**A**) Mean arterial pressure. (**B**) Mean pulmonary artery pressure. (**C**) Cardiac output. (**D**) Right atrial pressure. (**E**) Pulmonary artery occlusion pressure. (**F**) Norepinephrine. Mixed-effects models were used and the overall P value for the effect of position without body flexion from 0° to 90° is provided.


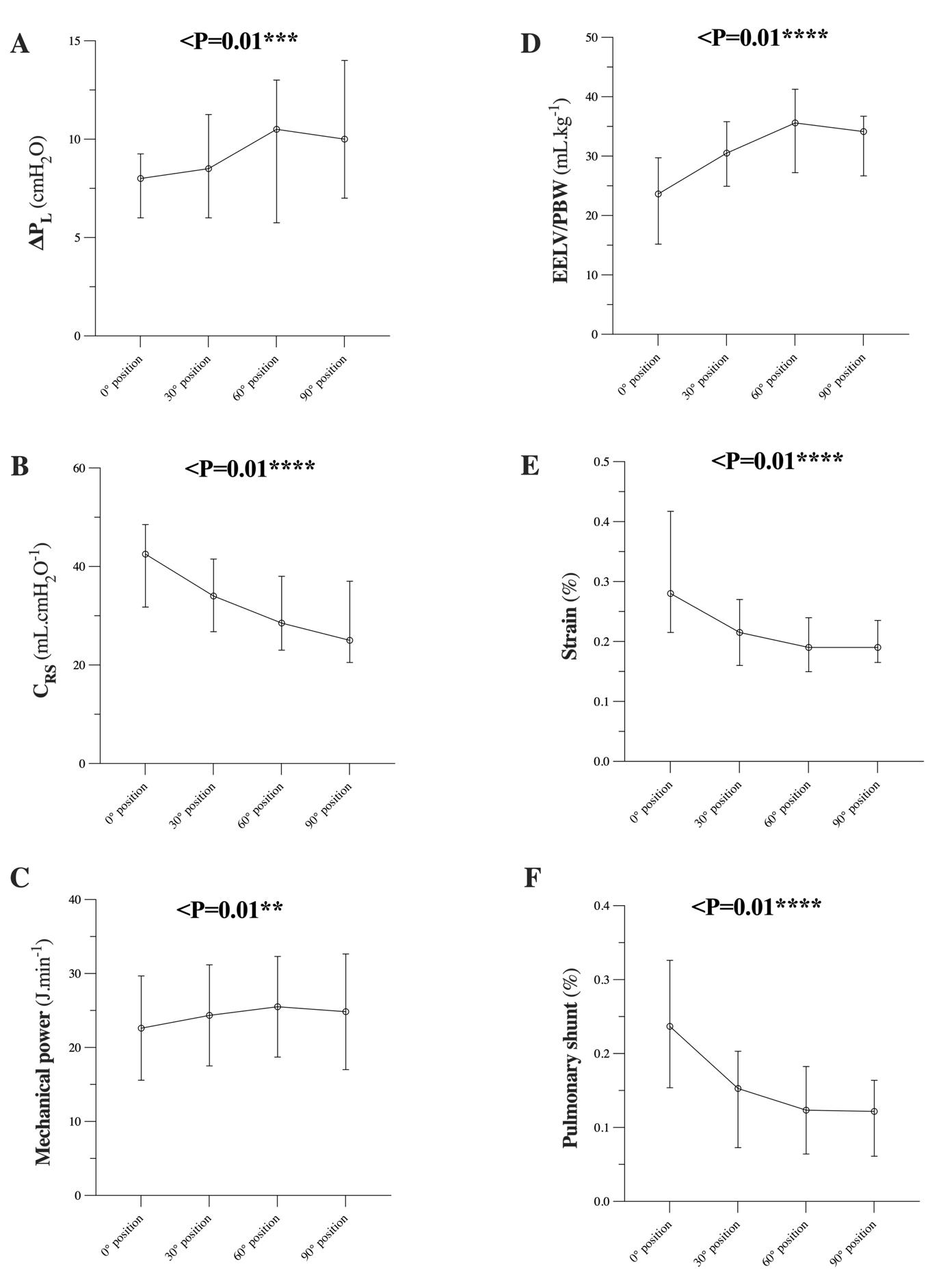


**Figure E5.** **Respiratory values evaluated at each position without body flexion, from 0° to 90°.** Values are reported as column with connected median and interquartile range. (**A**) Transpulmonary driving pressure (ΔP_L_), computed as the difference between end-inspiratory and end-expiratory transpulmonary pressures, as measured using an esophageal balloon catheter. (**B**) Static compliance of the respiratory system (C_RS_). (**C**) Mechanical power in the different study steps. (**D**) End-expiratory lung volume, as expressed in milliliters per kilogram of predicted body weight (EELV_PBW_). (**E**) Strain, computed as the tidal volume-to-EELV ratio. (**F**) Pulmonary shunt measured using the venous-to-arterial difference in oxygen concentrations. Mixed-effects models were used and the overall P value for the effect of position without body flexion from 0° to 90° is provided.


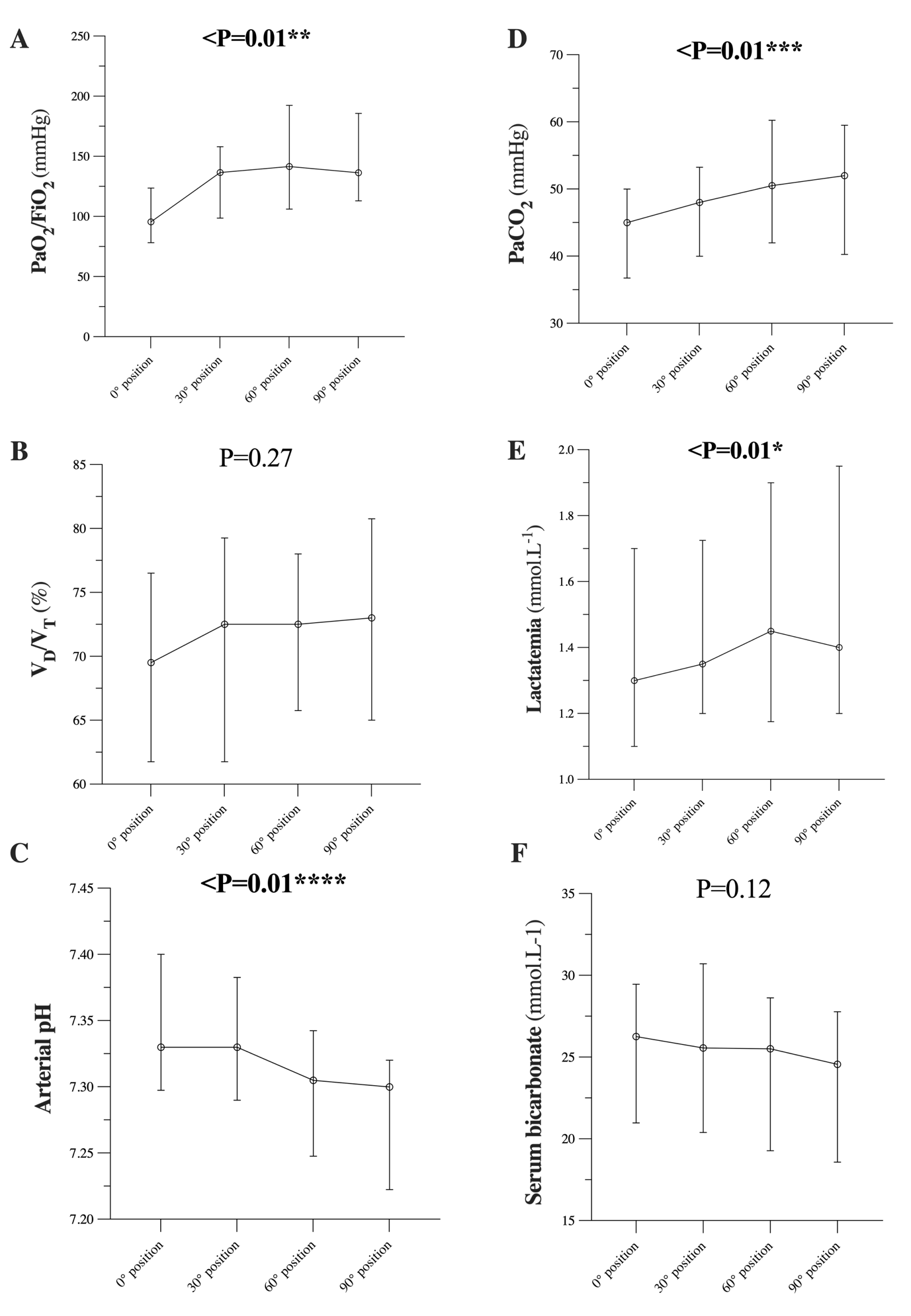


**Figure E6.** **Gasometrical values evaluated at each position without body flexion, from 0° to 90°.** Values are reported as column with connected median and interquartile range. (**A**) PaO_2_: arterial partial pressure of oxygen. FiO_2_: fraction of inspired oxygen. (**B**) V_D_/V_T_: dead-space fraction. (**C**) Arterial pH. (**D**) PaCO_2_: arterial partial pressure of carbon dioxide. (**E**) Lactatemia. (**F**) Serum bicarbonate. Mixed-effects models were used and the overall P value for the effect of position without body flexion from 0° to 90° is provided.

**
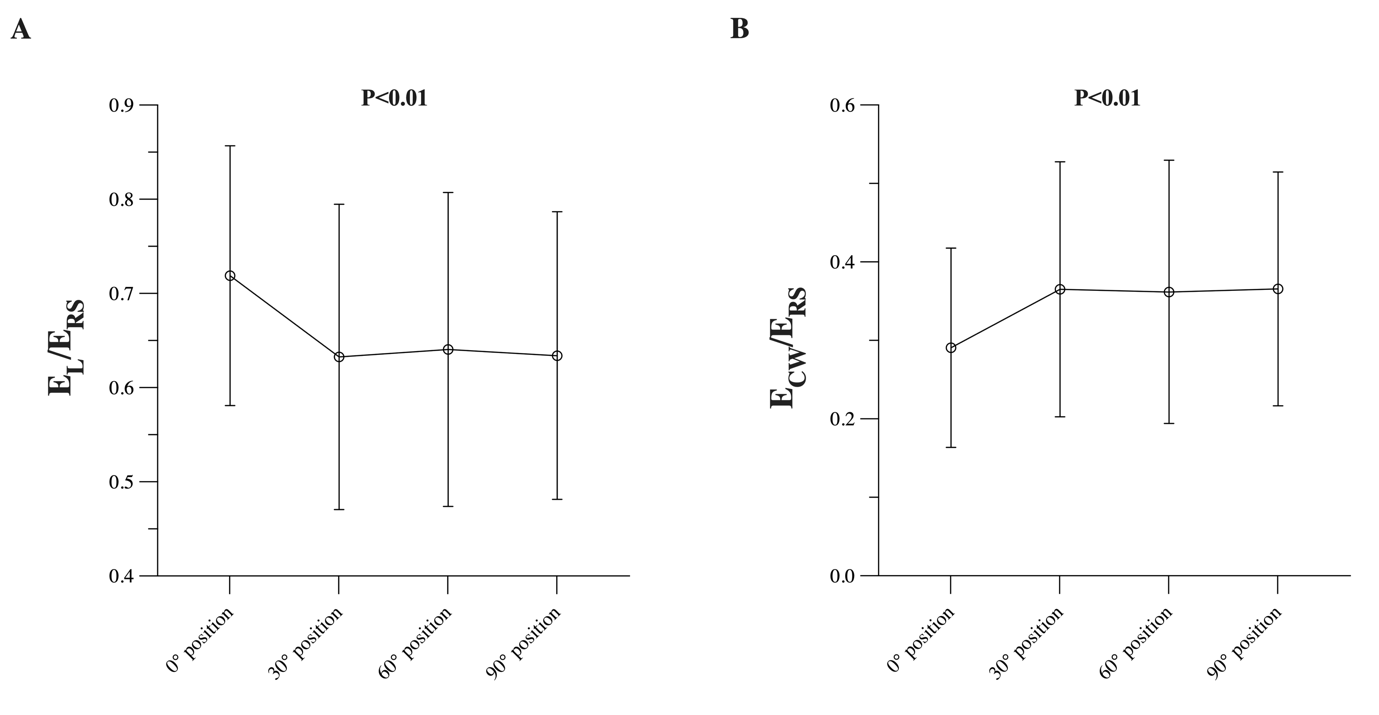
Figure E7.** **Relationship between lung elastance and total respiratory system elastance, and between chest wall elastance and respiratory system elastance evaluated at each position without body flexion, from 0° to 90°.** Values are reported as column with connected median and interquartile range. (**A**) E_L_/E_RS_: lung elastance and respiratory system elastance ratio. (**B**) E_CW_/E_RS_: dead-space fraction. Mixed-effects models were used and the overall P value for the effect of position without body flexion from 0° to 90° is provided.

**
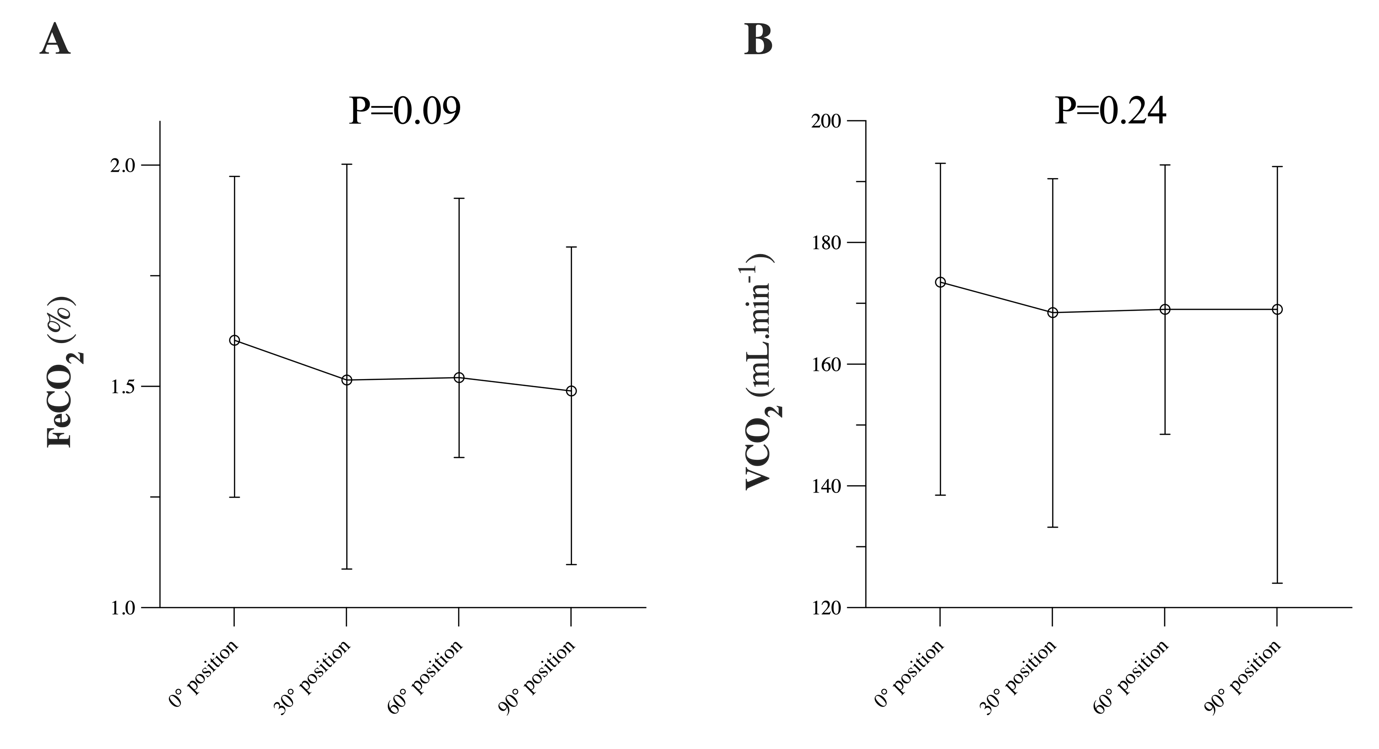
**

**Figure E8. FeCO_2_ and VCO_2_ values evaluated at each position without body flexion, from 0° to 90°.** Values are reported as column with connected median and interquartile range. (**A**) FeCO_2_: fraction of inspired carbon dioxide. (**B**) VCO_2_: volume of carbon dioxide eliminated per minute. Mixed-effects models were used and the overall P value for the effect of position without body flexion from 0° to 90° is provided.

**REFERENCES**

1. ARDS Definition Task Force, Ranieri VM, Rubenfeld GD, Thompson BT, Ferguson ND, Caldwell E, et al. Acute respiratory distress syndrome: the Berlin Definition. JAMA 2012;307:2526–2533.

2. Chiumello D, Cressoni M, Chierichetti M, Tallarini F, Botticelli M, Berto V, et al. Nitrogen washout/washin, helium dilution and computed tomography in the assessment of end expiratory lung volume. Critical Care 2008;12:R150.

3. Akoumianaki E, Maggiore SM, Valenza F, Bellani G, Jubran A, Loring SH, et al. The Application of Esophageal Pressure Measurement in Patients with Respiratory Failure. Am J Respir Crit Care Med 2014;189:520–531.

4. Vallet B, Blanloeil Y, Cholley B, Orliaguet G, Pierre S, Tavernier B, et al. Guidelines for perioperative haemodynamic optimization. Ann Fr Anesth Reanim 2013;32:e151-158.

5. Fenn WO, Rahn H, Otis AB. A theoretical study of the composition of the alveolar air at altitude. Am J Physiol 1946;146:637–653.

6. Long CL, Schaffel N, Geiger JW, Schiller WR, Blakemore WS. Metabolic response to injury and illness: estimation of energy and protein needs from indirect calorimetry and nitrogen balance. JPEN J Parenter Enteral Nutr 1979;3:452–456.

7. Berggren SM. The oxygen deficit of arterial blood caused by non-ventilating parts of the lung. Acta Physiologica Scandinavica 1942;11:4.

8. Chiumello D, Carlesso E, Cadringher P, Caironi P, Valenza F, Polli F, et al. Lung Stress and Strain during Mechanical Ventilation for Acute Respiratory Distress Syndrome. Am J Respir Crit Care Med 2008;178:346–355.

9. Gattinoni L, Tonetti T, Cressoni M, Cadringher P, Herrmann P, Moerer O, et al. Ventilator-related causes of lung injury: the mechanical power. Intensive Care Med 2016;42:1567–1575.

10. Siddiki H, Kojicic M, Li G, Yilmaz M, Thompson TB, Hubmayr RD, et al. Bedside quantification of dead-space fraction using routine clinical data in patients with acute lung injury: secondary analysis of two prospective trials. Crit Care 2010;14:R141.

11. Eldridge SM, Chan CL, Campbell MJ, Bond CM, Hopewell S, Thabane L, et al. CONSORT 2010 statement: extension to randomised pilot and feasibility trials. BMJ 2016;355:i5239.

12. Julious SA. Sample size of 12 per group rule of thumb for a pilot study. Pharmaceutical Statistics 2005;4:287–291.

13. Teare MD, Dimairo M, Shephard N, Hayman A, Whitehead A, Walters SJ. Sample size requirements to estimate key design parameters from external pilot randomised controlled trials: a simulation study. Trials 2014;15:264.

14. Browne RH. On the use of a pilot sample for sample size determination. Stat Med 1995;14:1933–1940.

15. Whitehead AL, Julious SA, Cooper CL, Campbell MJ. Estimating the sample size for a pilot randomised trial to minimise the overall trial sample size for the external pilot and main trial for a continuous outcome variable. Stat Methods Med Res 2016;25:1057–1073.

16. Durig A. Über die Grosse der Residualluft. Zentralblatt Physiologie 1903;258:67.

17. Fretschner R, Deusch H, Weitnauer A, Brunner JX. A simple method to estimate functional residual capacity in mechanically ventilated patients. Intensive Care Med 1993;19:372–376.

18. Olegård C, Söndergaard S, Houltz E, Lundin S, Stenqvist O. Estimation of functional residual capacity at the bedside using standard monitoring equipment: a modified nitrogen washout/washin technique requiring a small change of the inspired oxygen fraction. Anesth Analg 2005;101:206–212, table of contents.

19. Arias-Ortiz J, Vincent J-L. The pulmonary artery catheter. Curr Opin Crit Care 2023;29:231–235.

20. Bootsma IT, Boerma EC, de Lange F, Scheeren TWL. The contemporary pulmonary artery catheter. Part 1: placement and waveform analysis. J Clin Monit Comput 2022;36:5–15.

21. Bootsma IT, Boerma EC, Scheeren TWL, de Lange F. The contemporary pulmonary artery catheter. Part 2: measurements, limitations, and clinical applications. J Clin Monit Comput 2022;36:17–31.

22. Protections (OHRP) O for HR. Reviewing and Reporting Unanticipated Problems Involving Risks to Subjects or Others and Adverse Events: OHRP Guidance (2007). 2010;at <https://www.hhs.gov/ohrp/regulations-and-policy/guidance/reviewing-unanticipated-problems/index.html>.

23. Zeppenfeld K, Tfelt-Hansen J, de Riva M, Winkel BG, Behr ER, Blom NA, et al. 2022 ESC Guidelines for the management of patients with ventricular arrhythmias and the prevention of sudden cardiac death: Developed by the task force for the management of patients with ventricular arrhythmias and the prevention of sudden cardiac death of the European Society of Cardiology (ESC) Endorsed by the Association for European Paediatric and Congenital Cardiology (AEPC). European Heart Journal 2022;43:3997–4126.

24. Hindricks G, Potpara T, Dagres N, Arbelo E, Bax JJ, Blomström-Lundqvist C, et al. 2020 ESC Guidelines for the diagnosis and management of atrial fibrillation developed in collaboration with the European Association for Cardio-Thoracic Surgery (EACTS): The Task Force for the diagnosis and management of atrial fibrillation of the European Society of Cardiology (ESC) Developed with the special contribution of the European Heart Rhythm Association (EHRA) of the ESC. European Heart Journal 2021;42:373–498.

25. Soar J, Böttiger BW, Carli P, Couper K, Deakin CD, Djärv T, et al. European Resuscitation Council Guidelines 2021: Adult advanced life support. Resuscitation 2021;161:115–151.
